# Mendelian randomisation study of smoking exposure in relation to breast cancer risk

**DOI:** 10.1038/s41416-021-01432-8

**Published:** 2021-08-02

**Authors:** Hanla A. Park, Sonja Neumeyer, Kyriaki Michailidou, Manjeet K. Bolla, Qin Wang, Joe Dennis, Thomas U. Ahearn, Irene L. Andrulis, Hoda Anton-Culver, Natalia N. Antonenkova, Volker Arndt, Kristan J. Aronson, Annelie Augustinsson, Adinda Baten, Laura E. Beane Freeman, Heiko Becher, Matthias W. Beckmann, Sabine Behrens, Javier Benitez, Marina Bermisheva, Natalia V. Bogdanova, Stig E. Bojesen, Hiltrud Brauch, Hermann Brenner, Sara Y. Brucker, Barbara Burwinkel, Daniele Campa, Federico Canzian, Jose E. Castelao, Stephen J. Chanock, Georgia Chenevix-Trench, Christine L. Clarke, NBCS Collaborators, Anne-Lise Børresen-Dale, Grethe I. Grenaker Alnæs, Kristine K. Sahlberg, Lars Ottestad, Rolf Kåresen, Ellen Schlichting, Marit Muri Holmen, Toril Sauer, Vilde Haakensen, Olav Engebråten, Bjørn Naume, Alexander Fosså, Cecile E. Kiserud, Kristin V. Reinertsen, Åslaug Helland, Margit Riis, Jürgen Geisler, Don M. Conroy, Fergus J. Couch, Angela Cox, Simon S. Cross, Kamila Czene, Mary B. Daly, Peter Devilee, Thilo Dörk, Isabel dos-Santos-Silva, Miriam Dwek, Diana M. Eccles, A. Heather Eliassen, Christoph Engel, Mikael Eriksson, D. Gareth Evans, Peter A. Fasching, Henrik Flyger, Lin Fritschi, Montserrat García-Closas, José A. García-Sáenz, Mia M. Gaudet, Graham G. Giles, Gord Glendon, Mark S. Goldberg, David E. Goldgar, Anna González-Neira, Mervi Grip, Pascal Guénel, Eric Hahnen, Christopher A. Haiman, Niclas Håkansson, Per Hall, Ute Hamann, Sileny Han, Elaine F. Harkness, Steven N. Hart, Wei He, Bernadette A. M. Heemskerk-Gerritsen, John L. Hopper, David J. Hunter, ABCTB Investigators, Christine Clarke, Deborah Marsh, Rodney Scott, Robert Baxter, Desmond Yip, Jane Carpenter, Alison Davis, Nirmala Pathmanathan, Peter Simpson, Dinny Graham, Mythily Sachchithananthan, kConFab Investigators, David Amor, Lesley Andrews, Yoland Antill, Rosemary Balleine, Jonathan Beesley, Ian Bennett, Michael Bogwitz, Leon Botes, Meagan Brennan, Melissa Brown, Michael Buckley, Jo Burke, Phyllis Butow, Liz Caldon, Ian Campbell, Deepa Chauhan, Manisha Chauhan, Georgia Chenevix-Trench, Alice Christian, Paul Cohen, Alison Colley, Ashley Crook, James Cui, Margaret Cummings, Sarah-Jane Dawson, Anna DeFazio, Martin Delatycki, Rebecca Dickson, Joanne Dixon, Ted Edkins, Stacey Edwards, Gelareh Farshid, Andrew Fellows, Georgina Fenton, Michael Field, James Flanagan, Peter Fong, Laura Forrest, Stephen Fox, Juliet French, Michael Friedlander, Clara Gaff, Mike Gattas, Peter George, Sian Greening, Marion Harris, Stewart Hart, Nick Hayward, John Hopper, Cass Hoskins, Clare Hunt, Paul James, Mark Jenkins, Alexa Kidd, Judy Kirk, Jessica Koehler, James Kollias, Sunil Lakhani, Mitchell Lawrence, Geoff Lindeman, Lara Lipton, Liz Lobb, Graham Mann, Deborah Marsh, Sue Anne McLachlan, Bettina Meiser, Roger Milne, Sophie Nightingale, Shona O’Connell, Sarah O’Sullivan, David Gallego Ortega, Nick Pachter, Briony Patterson, Amy Pearn, Kelly Phillips, Ellen Pieper, Edwina Rickard, Bridget Robinson, Mona Saleh, Elizabeth Salisbury, Christobel Saunders, Jodi Saunus, Rodney Scott, Clare Scott, Adrienne Sexton, Andrew Shelling, Peter Simpson, Melissa Southey, Amanda Spurdle, Jessica Taylor, Renea Taylor, Heather Thorne, Alison Trainer, Kathy Tucker, Jane Visvader, Logan Walker, Rachael Williams, Ingrid Winship, Mary Ann Young, Agnes Jager, Anna Jakubowska, Esther M. John, Audrey Jung, Rudolf Kaaks, Pooja Middha Kapoor, Renske Keeman, Elza Khusnutdinova, Cari M. Kitahara, Linetta B. Koppert, Stella Koutros, Vessela N. Kristensen, Allison W. Kurian, James Lacey, Diether Lambrechts, Loic Le Marchand, Wing-Yee Lo, Jan Lubiński, Arto Mannermaa, Mehdi Manoochehri, Sara Margolin, Maria Elena Martinez, Dimitrios Mavroudis, Alfons Meindl, Usha Menon, Roger L. Milne, Taru A. Muranen, Heli Nevanlinna, William G. Newman, Børge G. Nordestgaard, Kenneth Offit, Andrew F. Olshan, Håkan Olsson, Tjoung-Won Park-Simon, Paolo Peterlongo, Julian Peto, Dijana Plaseska-Karanfilska, Nadege Presneau, Paolo Radice, Gad Rennert, Hedy S. Rennert, Atocha Romero, Emmanouil Saloustros, Elinor J. Sawyer, Marjanka K. Schmidt, Rita K. Schmutzler, Minouk J. Schoemaker, Lukas Schwentner, Christopher Scott, Mitul Shah, Xiao-Ou Shu, Jacques Simard, Ann Smeets, Melissa C. Southey, John J. Spinelli, Victoria Stevens, Anthony J. Swerdlow, Rulla M. Tamimi, William J. Tapper, Jack A. Taylor, Mary Beth Terry, Ian Tomlinson, Melissa A. Troester, Thérèse Truong, Celine M. Vachon, Elke M. van Veen, Joseph Vijai, Sophia Wang, Camilla Wendt, Robert Winqvist, Alicja Wolk, Argyrios Ziogas, Alison M. Dunning, Paul D. P. Pharoah, Douglas F. Easton, Wei Zheng, Peter Kraft, Jenny Chang-Claude

**Affiliations:** 1 Division of Cancer Epidemiology, German Cancer Research Center (DKFZ), Heidelberg, Germany; 2 Faculty of Medicine, University of Heidelberg, Heidelberg, Germany; 3 Biostatistics Unit, The Cyprus Institute of Neurology & Genetics, Nicosia, Cyprus; 4 Cyprus School of Molecular Medicine, The Cyprus Institute of Neurology & Genetics, Nicosia, Cyprus; 5 Centre for Cancer Genetic Epidemiology, Department of Public Health and Primary Care, University of Cambridge, Cambridge, UK; 6 Division of Cancer Epidemiology and Genetics, National Cancer Institute, National Institutes of Health, Department of Health and Human Services, Bethesda, MD USA; 7 Fred A. Litwin Center for Cancer Genetics, Lunenfeld-Tanenbaum Research Institute of Mount Sinai Hospital, Toronto, ON Canada; 8 Department of Molecular Genetics, University of Toronto, Toronto, ON Canada; 9 Department of Epidemiology, Genetic Epidemiology Research Institute, University of California Irvine, Irvine, CA USA; 10 N.N. Alexandrov Research Institute of Oncology and Medical Radiology, Minsk, Belarus; 11 Division of Clinical Epidemiology and Aging Research, German Cancer Research Center (DKFZ), Heidelberg, Germany; 12 Department of Public Health Sciences, and Cancer Research Institute, Queen’s University, Kingston, ON Canada; 13 Department of Cancer Epidemiology, Clinical Sciences, Lund University, Lund, Sweden; 14 Leuven Multidisciplinary Breast Center, Department of Oncology, Leuven Cancer Institute, University Hospitals Leuven, Leuven, Belgium; 15 Institute of Medical Biometry and Epidemiology, University Medical Center Hamburg-Eppendorf, Hamburg, Germany; 16 Institute of Biometry and Clinical Epidemiology, Charité –Universitätsmedizin Berlin, Berlin, Germany; 17 Department of Gynecology and Obstetrics, Comprehensive Cancer Center ER-EMN, University Hospital Erlangen, Friedrich-Alexander-University Erlangen-Nuremberg, Erlangen, Germany; 18 Centro de Investigación en Red de Enfermedades Raras (CIBERER), Madrid, Spain; 19 Human Cancer Genetics Programme, Spanish National Cancer Research Centre (CNIO), Madrid, Spain; 20 Institute of Biochemistry and Genetics, Ufa Federal Research Centre of the Russian Academy of Sciences, Ufa, Russia; 21 Department of Radiation Oncology, Hannover Medical School, Hannover, Germany; 22 Gynaecology Research Unit, Hannover Medical School, Hannover, Germany; 23 Copenhagen General Population Study, Herlev and Gentofte Hospital, Copenhagen University Hospital, Herlev, Denmark; 24 Department of Clinical Biochemistry, Herlev and Gentofte Hospital, Copenhagen University Hospital, Herlev, Denmark; 25 Faculty of Health and Medical Sciences, University of Copenhagen, Copenhagen, Denmark; 26 Dr. Margarete Fischer-Bosch-Institute of Clinical Pharmacology, Stuttgart, Germany; 27 iFIT-Cluster of Excellence, University of Tübingen, Tübingen, Germany; 28 German Cancer Consortium (DKTK), German Cancer Research Center (DKFZ), Partner Site Tübingen, Tübingen, Germany; 29 Division of Preventive Oncology, German Cancer Research Center (DKFZ) and National Center for Tumor Diseases (NCT), Heidelberg, Germany; 30 German Cancer Consortium (DKTK), German Cancer Research Center (DKFZ), Heidelberg, Germany; 31 Department of Gynecology and Obstetrics, University of Tübingen, Tübingen, Germany; 32 Molecular Epidemiology Group, C080, German Cancer Research Center (DKFZ), Heidelberg, Germany; 33 Molecular Biology of Breast Cancer, University Womens Clinic Heidelberg, University of Heidelberg, Heidelberg, Germany; 34 Department of Biology, University of Pisa, Pisa, Italy; 35 Genomic Epidemiology Group, German Cancer Research Center (DKFZ), Heidelberg, Germany; 36 Oncology and Genetics Unit, Instituto de Investigacion Sanitaria Galicia Sur (IISGS), Xerencia de Xestion Integrada de Vigo-SERGAS, Vigo, Spain; 37 Department of Genetics and Computational Biology, QIMR Berghofer Medical Research Institute, Brisbane, QLD Australia; 38 Westmead Institute for Medical Research, University of Sydney, Sydney, NSW Australia; 39 Centre for Cancer Genetic Epidemiology, Department of Oncology, University of Cambridge, Cambridge, UK; 40 Department of Laboratory Medicine and Pathology, Mayo Clinic, Rochester, MN USA; 41 Sheffield Institute for Nucleic Acids (SInFoNiA), Department of Oncology and Metabolism, University of Sheffield, Sheffield, UK; 42 Academic Unit of Pathology, Department of Neuroscience, University of Sheffield, Sheffield, UK; 43 Department of Medical Epidemiology and Biostatistics, Karolinska Institutet, Stockholm, Sweden; 44 Department of Clinical Genetics, Fox Chase Cancer Center, Philadelphia, PA USA; 45 Department of Pathology, Leiden University Medical Center, Leiden, The Netherlands; 46 Department of Human Genetics, Leiden University Medical Center, Leiden, The Netherlands; 47 Department of Non-Communicable Disease Epidemiology, London School of Hygiene and Tropical Medicine, London, UK; 48 School of Life Sciences, University of Westminster, London, UK; 49 Faculty of Medicine, University of Southampton, Southampton, UK; 50 Channing Division of Network Medicine, Department of Medicine, Brigham and Women’s Hospital and Harvard Medical School, Boston, MA USA; 51 Department of Epidemiology, Harvard T.H. Chan School of Public Health, Boston, MA USA; 52 Institute for Medical Informatics, Statistics and Epidemiology, University of Leipzig, Leipzig, Germany; 53 Division of Evolution and Genomic Sciences, School of Biological Sciences, Faculty of Biology, Medicine and Health, University of Manchester, Manchester Academic Health Science Centre, Manchester, UK; 54 North West Genomics Laboratory Hub, Manchester Centre for Genomic Medicine, St Mary’s Hospital, Manchester University NHS Foundation Trust, Manchester Academic Health Science Centre, Manchester, UK; 55 David Geffen School of Medicine, Department of Medicine Division of Hematology and Oncology, University of California at Los Angeles, Los Angeles, CA USA; 56 Department of Breast Surgery, Herlev and Gentofte Hospital, Copenhagen University Hospital, Herlev, Denmark; 57 School of Public Health, Curtin University, Perth, WA Australia; 58 Medical Oncology Department, Hospital Clínico San Carlos, Instituto de Investigación Sanitaria San Carlos (IdISSC), Centro Investigación Biomédica en Red de Cáncer (CIBERONC), Madrid, Spain; 59 Behavioral and Epidemiology Research Group, American Cancer Society, Atlanta, GA USA; 60 Cancer Epidemiology Division, Cancer Council Victoria, Melbourne, VIC Australia; 61 Centre for Epidemiology and Biostatistics, Melbourne School of Population and Global Health, The University of Melbourne, Melbourne, VIC Australia; 62 Precision Medicine, School of Clinical Sciences at Monash Health, Monash University, Clayton, VIC Australia; 63 Department of Medicine, McGill University, Montréal, QC Canada; 64 Division of Clinical Epidemiology, Royal Victoria Hospital, McGill University, Montréal, QC Canada; 65 Department of Dermatology, Huntsman Cancer Institute, University of Utah School of Medicine, Salt Lake City, UT USA; 66 Department of Surgery, Oulu University Hospital, University of Oulu, Oulu, Finland; 67 Cancer & Environment Group, Center for Research in Epidemiology and Population Health (CESP), INSERM, University Paris-Sud, University Paris-Saclay, Villejuif, France; 68 Center for Familial Breast and Ovarian Cancer, Faculty of Medicine and University Hospital Cologne, University of Cologne, Cologne, Germany; 69 Center for Integrated Oncology (CIO), Faculty of Medicine and University Hospital Cologne, University of Cologne, Cologne, Germany; 70 Department of Preventive Medicine, Keck School of Medicine, University of Southern California, Los Angeles, CA USA; 71 Institute of Environmental Medicine, Karolinska Institutet, Stockholm, Sweden; 72 Department of Oncology, Södersjukhuset, Stockholm Sweden; 73 Molecular Genetics of Breast Cancer, German Cancer Research Center (DKFZ), Heidelberg, Germany; 74 Division of Informatics, Imaging and Data Sciences, Faculty of Biology, Medicine and Health, University of Manchester, Manchester Academic Health Science Centre, Manchester, UK; 75 Nightingale & Genesis Prevention Centre, Wythenshawe Hospital, Manchester University NHS Foundation Trust, Manchester, UK; 76 NIHR Manchester Biomedical Research Unit, Manchester University NHS Foundation Trust, Manchester Academic Health Science Centre, Manchester, UK; 77 Department of Health Sciences Research, Mayo Clinic, Rochester, MN USA; 78 Department of Medical Oncology, Erasmus MC Cancer Institute, Rotterdam, The Netherlands; 79 Program in Genetic Epidemiology and Statistical Genetics, Harvard T.H. Chan School of Public Health, Boston, MA USA; 80 Nuffield Department of Population Health, University of Oxford, Oxford, UK; 81 Department of Genetics and Pathology, Pomeranian Medical University, Szczecin, Poland; 82 Independent Laboratory of Molecular Biology and Genetic Diagnostics, Pomeranian Medical University, Szczecin, Poland; 83 Department of Epidemiology and Population Health, Stanford University School of Medicine, Stanford, CA USA; 84 Department of Medicine, Division of Oncology, Stanford Cancer Institute, Stanford University School of Medicine, Stanford, CA USA; 85 Division of Molecular Pathology, The Netherlands Cancer Institute - Antoni van Leeuwenhoek Hospital, Amsterdam, The Netherlands; 86 Department of Genetics and Fundamental Medicine, Bashkir State University, Ufa, Russia; 87 Radiation Epidemiology Branch, Division of Cancer Epidemiology and Genetics, National Cancer Institute, Bethesda, MD USA; 88 Department of Surgical Oncology, Family Cancer Clinic, Erasmus MC Cancer Institute, Rotterdam, The Netherlands; 89 Medical Genetics, Oslo University Hospital and University of Oslo, Oslo, Norway; 90 Department of Computational and Quantitative Medicine, City of Hope, Duarte, CA USA; 91 City of Hope Comprehensive Cancer Center, City of Hope, Duarte, CA USA; 92 VIB Center for Cancer Biology, Leuven, Belgium; 93 Laboratory for Translational Genetics, Department of Human Genetics, University of Leuven, Leuven, Belgium; 94 Epidemiology Program, University of Hawaii Cancer Center, Honolulu, HI USA; 95 University of Tübingen, Tübingen, Germany; 96 Translational Cancer Research Area, University of Eastern Finland, Kuopio, Finland; 97 Institute of Clinical Medicine, Pathology and Forensic Medicine, University of Eastern Finland, Kuopio, Finland; 98 Biobank of Eastern Finland, Kuopio University Hospital, Kuopio, Finland; 99 Department of Clinical Science and Education, Södersjukhuset, Karolinska Institutet, Stockholm, Sweden; 100 Moores Cancer Center, University of California San Diego, La Jolla, CA USA; 101 Department of Family Medicine and Public Health, University of California San Diego, La Jolla, CA USA; 102 Department of Medical Oncology, University Hospital of Heraklion, Heraklion, Greece; 103 Department of Gynecology and Obstetrics, University of Munich, Campus Großhadern, Munich Germany; 104 MRC Clinical Trials Unit at UCL, Institute of Clinical Trials & Methodology, University College London, London, UK; 105 Department of Obstetrics and Gynecology, Helsinki University Hospital, University of Helsinki, Helsinki, Finland; 106 Clinical Genetics Research Lab, Department of Cancer Biology and Genetics, Memorial Sloan Kettering Cancer Center, New York, NY USA; 107 Clinical Genetics Service, Department of Medicine, Memorial Sloan Kettering Cancer Center, New York, NY USA; 108 Department of Epidemiology, Gillings School of Global Public Health and UNC Lineberger Comprehensive Cancer Center, University of North Carolina at Chapel Hill, Chapel Hill, NC USA; 109 Genome Diagnostics Program, IFOM - the FIRC Institute of Molecular Oncology, Milan, Italy; 110 Research Centre for Genetic Engineering and Biotechnology ‘Georgi D, Efremov’, MASA, Skopje, Republic of North Macedonia; 111 Unit of Molecular Bases of Genetic Risk and Genetic Testing, Department of Research, Fondazione IRCCS Istituto Nazionale dei Tumori (INT), Milan, Italy; 112 Clalit National Cancer Control Center, Carmel Medical Center and Technion Faculty of Medicine, Haifa, Israel; 113 Medical Oncology Department, Hospital Universitario Puerta de Hierro, Madrid, Spain; 114 Department of Oncology, University Hospital of Larissa, Larissa, Greece; 115 School of Cancer & Pharmaceutical Sciences, Comprehensive Cancer Centre, Guy’s Campus, King’s College London, London, UK; 116 Division of Psychosocial Research and Epidemiology, The Netherlands Cancer Institute - Antoni van Leeuwenhoek hospital, Amsterdam, The Netherlands; 117 Center for Molecular Medicine Cologne (CMMC), Faculty of Medicine and University Hospital Cologne, University of Cologne, Cologne, Germany; 118 Division of Genetics and Epidemiology, The Institute of Cancer Research, London, UK; 119 Department of Gynaecology and Obstetrics, University Hospital Ulm, Ulm, Germany; 120 Division of Epidemiology, Department of Medicine, Vanderbilt Epidemiology Center, Vanderbilt-Ingram Cancer Center, Vanderbilt University School of Medicine, Nashville, TN USA; 121 Genomics Center, Centre Hospitalier Universitaire de Québec – Université Laval Research Center, Québec City, QC Canada; 122 Department of Surgical Oncology, University Hospitals Leuven, Leuven, Belgium; 123 Department of Clinical Pathology, The University of Melbourne, Melbourne, VIC Australia; 124 Population Oncology, BC Cancer, Vancouver, BC Canada; 125 School of Population and Public Health, University of British Columbia, Vancouver, BC Canada; 126 Division of Breast Cancer Research, The Institute of Cancer Research, London, UK; 127 Epidemiology Branch, National Institute of Environmental Health Sciences, NIH, Research Triangle Park, NC USA; 128 Epigenetic and Stem Cell Biology Laboratory, National Institute of Environmental Health Sciences, NIH, Research Triangle Park, NC USA; 129 Department of Epidemiology, Mailman School of Public Health, Columbia University, New York, NY USA; 130 Institute of Cancer and Genomic Sciences, University of Birmingham, Birmingham, UK; 131 Wellcome Trust Centre for Human Genetics and Oxford NIHR Biomedical Research Centre, University of Oxford, Oxford, UK; 132 Department of Health Science Research, Division of Epidemiology, Mayo Clinic, Rochester, MN USA; 133 Laboratory of Cancer Genetics and Tumor Biology, Cancer and Translational Medicine Research Unit, Biocenter Oulu, University of Oulu, Oulu, Finland; 134 Laboratory of Cancer Genetics and Tumor Biology, Northern Finland Laboratory Centre Oulu, Oulu, Finland; 135 Department of Surgical Sciences, Uppsala University, Uppsala, Sweden; 136 Cancer Epidemiology Group, University Cancer Center Hamburg (UCCH), University Medical Center Hamburg-Eppendorf, Hamburg, Germany; 137 Department of Cancer Genetics, Institute for Cancer Research, Oslo University Hospital-Radiumhospitalet, Oslo, Norway; 138 Institute of Clinical Medicine, Faculty of Medicine, University of Oslo, Oslo, Norway; 139 Department of Research, Vestre Viken Hospital, Drammen, Norway; 140 Section for Breast- and Endocrine Surgery, Department of Cancer, Division of Surgery, Cancer and Transplantation Medicine, Oslo University Hospital-Ullevål, Oslo, Norway; 141 Department of Radiology and Nuclear Medicine, Oslo University Hospital, Oslo, Norway; 142 Department of Pathology, Akershus University Hospital, Lørenskog, Norway; 143 Department of Tumor Biology, Institute for Cancer Research, Oslo University Hospital, Oslo, Norway; 144 Department of Oncology, Division of Surgery, Cancer and Transplantation Medicine, Oslo University Hospital-Radiumhospitalet, Oslo, Norway; 145 National Advisory Unit on Late Effects after Cancer Treatment, Oslo University Hospital-Radiumhospitalet, Oslo, Norway; 146 Department of Oncology, Akershus University Hospital, Lørenskog, Norway; 147 Breast Cancer Research Consortium, Oslo University Hospital, Oslo, Norway; 148 Australian Breast Cancer Tissue Bank, Westmead Institute for Medical Research, University of Sydney, Sydney, NSW Australia; 149 Research department, Peter MacCallum Cancer Center, Melbourne, VIC Australia; 150 Sir Peter MacCallum Department of Oncology, The University of Melbourne, Melbourne, VIC Australia

**Keywords:** Breast cancer, Risk factors

## Abstract

**Background:**

Despite a modest association between tobacco smoking and breast cancer risk reported by recent epidemiological studies, it is still equivocal whether smoking is causally related to breast cancer risk.

**Methods:**

We applied Mendelian randomisation (MR) to evaluate a potential causal effect of cigarette smoking on breast cancer risk. Both individual-level data as well as summary statistics for 164 single-nucleotide polymorphisms (SNPs) reported in genome-wide association studies of lifetime smoking index (LSI) or cigarette per day (CPD) were used to obtain MR effect estimates. Data from 108,420 invasive breast cancer cases and 87,681 controls were used for the LSI analysis and for the CPD analysis conducted among ever-smokers from 26,147 cancer cases and 26,072 controls. Sensitivity analyses were conducted to address pleiotropy.

**Results:**

Genetically predicted LSI was associated with increased breast cancer risk (OR 1.18 per SD, 95% CI: 1.07–1.30, *P* = 0.11 × 10^–2^), but there was no evidence of association for genetically predicted CPD (OR 1.02, 95% CI: 0.78–1.19, *P* = 0.85). The sensitivity analyses yielded similar results and showed no strong evidence of pleiotropic effect.

**Conclusion:**

Our MR study provides supportive evidence for a potential causal association with breast cancer risk for lifetime smoking exposure but not cigarettes per day among smokers.

## Background

Breast cancer is the most common cancer in women, representing approximately one-quarter of all cancers diagnosed in women worldwide.^1^ Besides well-established risk factors for breast cancer, tobacco smoking has been widely studied as a potential risk factor for breast cancer since it is also a leading modifiable risk factor for cancers at sites not directly reached by tobacco smoke.^2,3^ Carcinogens associated with tobacco smoke include polycyclic aromatic hydrocarbons, aromatic amines and *N*-nitrosamines.^4^ It is biologically plausible that tobacco smoking may affect risk of breast cancer since metabolites of lipophilic tobacco-associated carcinogens have been detected in breast adipose tissue,^5,6^ and specific DNA adducts as well as p53 gene mutations are found in the breast cancer tissue of smokers.^7–9^

Based on the experimental and epidemiologic findings, evidence is insufficient to establish a causal relationship between tobacco smoking and breast cancer risk.^2,10^ Some of the inconsistencies in findings could be attributed to a potential dual effect of smoking on breast cancer.^11^ The anti-oestrogenic effect of smoking may attenuate or mask the carcinogenic effects.^12^ In a recent pooled analysis of 14 prospective cohort studies, adjusting for potential confounding by alcohol intake as well as body mass index (BMI), education, reproductive factors and other risk factors, a modest association was found between smoking and breast cancer risk.^13^ There was also an increased risk with longer duration of smoking prior to first birth, particularly for oestrogen receptor-positive tumours. It is however difficult to establish causation based on these observational studies.

Mendelian randomisation (MR) has been increasingly used to strengthen causal inference in observational studies and under certain assumptions is less vulnerable to residual confounding, reverse causation and selection bias.^14^ MR uses genetic variants such as single-nucleotide polymorphisms (SNPs) associated with an exposure of interest as an instrumental variable (IV), which is robust if three assumptions are met: (1) the genetic variants are casually associated with the exposure, (2) the variants are not associated with known or potential confounders for the exposure–outcome relationships and (3) the variants are associated with outcome only via the exposure of interest and not through other pathways.^15,16^ Given the unclear causal nature of the findings in observational studies, we conducted a MR analysis to investigate the association between smoking traits and breast cancer risk.

## Methods

### Study population

We used data from 81 studies participating in the Breast Cancer Association Consortium (BCAC), including 108,420 cases and 87,681 controls of European ancestry. Genotyping was performed using two custom-made genotyping arrays: OncoArray in 68,242 invasive breast cancer cases and 52,367 controls (https://epi.grants.cancer.gov/oncoarray/)^17^ and iCOGS arrays in 40,178 cases and 35,314 controls (http://ccge.medschl.cam.ac.uk/research/consortia/icogs/).^18^ Genotype data were imputed based on the 1000 Genomes project Phase 3 as the reference panel using the programme IMPUTE2.^19^ SNPs with high imputation quality (imputation *r*^2^ > 0.5) were included. Overlapping participants between datasets were excluded from the iCOGS dataset as the OncoArray provides a better genomic coverage than the iCOGS array. Demographic and epidemiologic data were harmonised across BCAC sites based on a standardised protocol and derived with respect to a reference date, which was date at diagnosis for cases and date at interview for controls. For controls and cases from the nested case-control studies, data from the baseline interview were considered, or if available, follow-up information. Chosen characteristics of the two datasets, including self-reported smoking behaviours (e.g., smoking status, smoking heaviness and smoking duration, including age at smoking initiation and lifetime smoking exposure) are shown in Supplementary Table 1. Ethics approval was obtained from the relevant institutional review boards for all BCAC studies, and all participants provided written informed consent.

### Selection of SNPs associated with smoking exposure

Exposure variables were selected based on the availability of associated genetic variants to reflect smoking exposure. We included two quantitative smoking behaviour-related traits, cigarettes per day (CPD; average number of cigarettes smoked per day by ever-smokers) and lifetime smoking index (LSI; composite score that captures lifetime smoking exposure by taking into account smoking status as well as smoking duration, heaviness and cessation in ever-smokers).^20^ The recent GWAS of cigarettes per day from GSCAN consortium identified 55 conditionally independent genome-wide significant SNPs, explaining 1.09% of the variance in a sample of 337,334 ever-smokers of European ancestry.^21^ For LSI, 126 significantly associated SNPs, 0.36% explained variance in LSI, were identified based on a sample of 426,690 individuals (never-smokers and ever-smokers) of European ancestry from the UK biobank.^20^ The genetic scores of the two smoking behaviour-related traits were reported to be associated with significantly higher risks of lung cancer.^20,22^

For our analysis, SNPs were selected if they were reported to be associated at genome-wide significance level (*P* ≤ 5 × 10^−8^) and had a minor allele frequency (MAF) above or equal 1%. For each behaviour phenotype, we filtered the list of behaviour-associated SNPs so that the remaining SNPs were not in linkage disequilibrium (LD) (*r*^2^ > 0.1). For CPD, the SNP with lowest *P* value (rs10519203) of the correlated SNPs (rs12438181, rs28438420, rs72740955, rs146009840, rs28681284, rs8040868, *r*^2^ > 0.01) was retained. One SNP (rs4886550) was not available in our data and without any proxy SNPs (LD, *r*^2^ > 0.8). Given that alcohol consumption is a recognised confounder of the association between smoking and breast cancer risk,^23–25^ we excluded nine SNPs (three of CPD, six of LSI, respectively) correlated with any alcohol consumption-associated SNPs (*r*^2^ > 0.1) from recent large-scale GWAS (drinks per week, *P* ≤ 5 × 10^−8^).^21^ After exclusions, we included a total of 164 variants associated with one of the smoking traits; 44 and 120 variants, respectively, for CPD and LSI (Supplementary Table 2). No variants overlapped between the two smoking traits.

### Statistical methods

#### Statistical power

Power calculations were conducted to estimate the magnitude of effects detectable with our study size assuming 5% α level and an *R*^2^ of 0.0109 for CPD and *R*^2^ of 0.0036 for LSI, which correspond to the variance in each smoking behaviour explained by the SNPs used for this analysis. Power calculations were performed using an online tool available at http://cnsgenomics.com/shiny/mRnd/.^26^ Detailed power calculations for all outcomes (invasive breast cancer, ER-positive and ER-negative) to detect different odd ratios are shown in Supplementary Table 3.

#### wPGS-based analyses

For our primary analysis, we generated weighted polygenic scores (wPGSs) using individual-level data of BCAC participants as follow: wPGS =$\mathop {\sum }\nolimits_{i = 1}^n \beta _{gx} \ast \alpha _i$, which is the sum of the effect allele dosage (α_i_) (ranging from 0 to 2) for each SNP weighted by the β-coefficient *β*_*gx*_ for the effect of the genetic variant (*g*) related to the smoking quality or/and duration (CPD, and LSI) (*x*) (Supplementary Table 2).^20,21^ The wPGSs of LSI was weakly correlated with wPGSs of CPD (0.17 in iCOGS and 0.03 in OncoArray) in ever-smokers. Analysis of LSI was conducted using all participants (including ever- and never-smokers), whereas that of CPD was performed solely in ever-smokers, and based on 26,147 cases (7342 for iCOGS, 18,805 for OncoArray) and 26,072 controls (8489 for iCOGS, 17,583 for OncoArray). These inclusion criteria correspond to those used in the GWAS studies that identified the SNPs for the smoking traits.^20,21^

Association analysis of the wPGSs with breast cancer risk using logistic regression was performed using fixed effect meta-analyses combining iCOGS and OncoArray results based on heterogeneity evaluated by Cochran’s Q statistics. The basic model (Model 1) was adjusted for age (continuous), principal components (PCs) of genetic ancestry (first ten PCs for iCOGS and OncoArray, separately) and study site, as previously described.^27^ In order to assess if the genetic instrument is independent of established risk factors for breast cancer, associations of wPGSs with selected breast cancer risk factors were assessed by linear regression for continuous variables and logistic regression for categorical variables (Supplementary Table 4). We adjusted for the risk factors that were associated with wPGSs of at least one of the smoking traits in Model 2. We additionally adjusted for alcohol assumption, a well-known confounder of the association between breast cancer risk and smoking, separately in Model 3 because of the large amount of missing data. Participants with missing covariables were excluded from all of the analyses.

Stratified analysis was performed to assess potential differences in associations with breast cancer risk by menopausal status (pre-, postmenopausal women) adjusting for age, study site and top ten PCs. Heterogeneity was tested employing the likelihood ratio test (LRT) for evaluating the multiplicative interaction terms in nested models. Polytomous regression was used to estimate the association according to oestrogen receptor (ER) status.

Scaling was applied to convert the wPGSs for CPD into meaningful units through dividing them by linear regression coefficients of self-reported CPD (0.35 per pack of cigarettes per day). The regression coefficient of CPD was derived from a meta-analysis of iCOGS and OncoArray data on smoking behaviours among 26,072 among ever-smoker controls (8490 and 17,703 controls of iCOGS and OncoArray, respectively).

#### Two-sample MR analyses

Five different two-sample MR methods using summary association data were applied: inverse-variance weighted (IVW),^28^ MR Egger,^29^ weighted median,^30^ weighted mode,^31^ and robust adjusted profile score (RAPS).^32^ Each of these methods makes slightly different assumptions about the nature of pleiotropy and therefore a roughly consistent point estimate across the multiple methods provides the strongest evidence of causal inference.^28^ The IVW method was implemented since the instruments consisted of multiple SNPs.^33^ Multivariable MR methods^34^ were conducted also using summary association data from GWASs of alcohol consumption (drinks per week),^21^ body mass index (BMI) among females^35^ and education attainment.^36^ To produce valid results, the IVW method requires that all instruments are associated with the exposure of interest (relevance assumption), but neither directly with the outcome of interest (only via the exposure; exclusion restriction) nor any confounders of the relationship between the exposure and the outcome (independence assumption).^37^ The intercept from MR-Egger regression is a statistical test for horizontal pleiotropy, whereas the slope can be interpreted as the smoking behaviour effect on breast cancer adjusted for horizontal pleiotropy.^29^ This method assumes however that the pleiotropic effects are independent of the instrument strength (InSIDE assumption). The weighted median estimator provides a valid causal estimate when at least half of the instruments are valid.^30^ The estimate from the weighted-mode analysis is valid when the largest group of instruments with consistent MR estimates is valid.^31^ MR-RAPS test extends the basic IVW random-effects approach by making the weight each variant receives in the analysis a function of the causal effect and the precision of the SNP-exposure association.^32^ The MR pleiotropy residual sum and outlier test (MR-PRESSO) was also implemented to identify outlying genetic variants and analyses were re-run after excluding these variants.^38^

All two-sample MR analyses using summary association data were performed with respect to three cancer susceptibility phenotypes: overall breast cancer (108,067 cases/88,386 controls) as well as oestrogen receptor (ER)-positive (70,435 cases) and ER-negative tumours (17,365 cases). Due to the nature of summary-level data, the analyses of both LSI and CPD were conducted using all samples regardless of smoking status.

R version 3.4.3 was used to conduct analyses. R package “Mendelian randomisation”, “mr_raps” and “MR-PRESSO” were used for two-sample MR analysis. All tests were considered at the 0.05 level of significance.

## Results

There was an association of wPGS for LSI with increased invasive breast cancer risk (OR per SD 1.18, 95% CI: 1.07–1.30, *P* = 0.11 × 10^–2^) whereas little evidence was found for an association between wPGSs for CPD and invasive breast cancer (OR 1.02 per pack of cigarettes per day, 95% CI: 0.78–1.19, *P* = 0.85) after adjustment for age and study (Model 1) (Table 1). Several breast cancer risk factors were associated with wPGSs of one of the smoking traits (CPD or/and LSI), including ever breastfeeding, menopausal status, age at menopause, BMI, age at first live birth, parity and education level (Supplementary Table 4). Adjustment for all of the identified risk factors did not change the association substantially (Model 2) (wPGSs for LSI, OR per SD 1.24, 95% CI: 1.06–1.45, *P* = 0.60 × 10^−2^) (Table 1). The point estimate of association between wPGS for LSI and invasive breast cancer risk remained unchanged after additional adjustment for alcohol consumption although imprecisely estimated (i.e. wide confidence intervals) (Model 3) (OR per SD 1.13, 95% CI: 0.86–1.49, *P* = 0.39). The association between wPGSs for CPD and invasive breast cancer did not change after adjustments.

**Table 1:** Associations of genetic risk scores for cigarette smoke exposure-related traits with breast cancer risk: results from Mendelian randomisation analysis.

	CPD				LSI			
	No. of cases/controls	OR^d^	95% CI	*P*	No. of cases/controls	OR^e^	95% CI	*P*
Model 1^a^	26,147/26,072	1.02	0.78–1.19	0.85	108,420/87,681	1.18	1.07–1.30	1.1 × 10^−3^
Model 2^b^	7360/7168	0.98	0.74–1.33	0.95	17,936/16,654	1.24	1.06–1.45	6.0 × 10^−3^
Model 3^c^	2892/2754	0.66	0.36–1.19	0.16	7716/7028	1.13	0.86–1.49	0.39

*No.* number, *CPD* cigarettes per day, *LSI* lifetime smoking index, *OR* odds ratio per year, *CI* confidence interval, P *P* value, *BMI* body mass index.

^a^Adjusted for age, sex and top ten PCs. *P* value for heterogeneity between iCOGS and OncoArray data of cigarettes per day and lifetime smoking index are 0.60, 0.09, respectively; ^b^in addition to adjustment of Model 1, additionally adjusted for ever breastfeeding, postmenopausal status, age at menopause, BMI, age at first live birth, parity and education level. *P* value for heterogeneity between iCOGS and OncoArray data of cigarettes per day and lifetime smoking index are 0.96, 0.37, respectively; ^c^in addition to adjustment of Model 2, additionally adjusted for alcohol assumption (glasses per day) *P* value for heterogeneity between iCOGS and OncoArray data of cigarettes per day and lifetime smoking index are 0.80, 0.66 respectively; ^d^OR per pack of cigarettes per day; ^e^OR per standard deviation.

There was no evidence for effect heterogeneity of the associations of LSI and CPD with breast cancer risk according to ER status or menopausal status (Supplementary Fig. 1).

Using IVW random-effects analysis, positive associations of genetically predicted LSI were found for overall breast cancer risk (OR per SD 1.14, 95% CI: 1.02–1.28, *P* = 0.02) and breast cancers according to ER status (OR per SD 1.14, 95% CI: 1.00–1.30, *P* = 0.04, for ER-positive and OR per SD 1.14, 95% CI: 0.95–1.37, *P* = 0.17, for ER-negative tumours) (Table 2, Supplementary Table 5 and Supplementary Figs. 2–4). There was no indication of horizontal pleiotropy based on the MR-Egger intercept test for any outcomes. The point estimates of associations were consistent across the different methods although the results based on MR-Egger regression, and weighted mode method were imprecisely estimated (i.e. wide confidence intervals). They remained substantially unchanged after multivariable adjustment for alcohol consumption, BMI and education. The MR-PRESSO analysis revealed three outliers for LSI. Removal of an outlier (rs2867112) with respect to risk for overall breast cancer and ER-negative cancer did not change the associations. No outlier was observed with ER-positive tumour (Table 2, Supplementary Table 5 and Supplementary Figs. 2–4).

**Table 2:** Association of cigarette smoke with overall breast cancer risk: results from the two-sample Mendelian randomisation using summary statistics.

	CPD			LSI		
	OR^i^	95% CI	*P*	OR^i^	95% CI	*P*
IVW summary statistics^a^	1.02	0.89–1.17	0.74	1.14	1.02–1.28	2.5 × 10^−02^
Multivariable IVW summary statistics^a,b^	1.05	0.92–1.20	0.49	1.38	1.08–1.76	8.9 × 10^−03^
Estimate using IVW after outlier corrected^a,c^	1.01	0.88–1.16	0.87	1.17	1.05–1.31	6.2 × 10^−03^
MR-Egger regression^a,d^	0.98	0.77–1.24	0.84	1.39	0.88–2.20	0.15
MR multivariable Egger regression^a,b,e^	1.03	0.82–1.29	0.82	1.36	0.88–2.11	0.17
MR weighted median estimator^a,f^	1.06	0.93–1.20	0.40	1.17	1.02–1.33	2.1 × 10^−02^
MR robust adjusted profile score estimator^a,g^	1.02	0.94–1.12	0.57	1.44	1.32 0 1.58	4.7 × 10^−15^
MR weighted mode^a,h^	1.01	0.89–1.15	0.84	1.23	0.86–1.75	0.26

*OR* odd ratio, *MR* Mendelian randomisation, *IVW* inverse-variance weighted, *CI* confidential interval, *CPD* cigarettes per day, *LSI* lifetime smoking index, *RAPS* robust adjusted profile score.

All two-sample MR analyses using summary-level data were performed in all samples regardless of smoking status (108,067 overall breast cancer cases/88,386 controls); ^a^estimate derived using summary statistics (28); ^b^multivariable analysis after adjusting for genetically predicted alcohol consumption (drinks per week), body mass index and education attainment by using summary-level data from GWAS outcome (alcohol assumption (21), body mass index (BMI) among female (35) and education attainment (36); ^c^the MR pleiotropy residual sum and outlier test (MR-PRESSO) was implemented to identify outlying genetic variants (rs11940255, rs1737894 and rs73229090 for CPD; rs2867112 for LSI) and analyses were re-run after excluding these variants (38); ^d^the MR-Egger intercept yielded no indication of strong pleiotropic effects (CPD: *β*_0_ = 0.24E-04, *P* = 0.99; LSI: *β*_0_ = −3.1E-03, *P* = 0.37); ^e^the multivariable MR-Egger intercept yielded no indication of strong pleiotropic effects (CPD: *β*_0_ = 6.88E-04, *P* = 0.83; LSI: *β*_0_ = 3.2E-04, *P* = 0.93); ^f^estimates derived using weighted median estimator approach, where 50% of the variants included in each genetic instrument are assumed to be invalid (30); ^g^estimates derived using Mendelian randomisation robust adjusted profile score (MR-RAPS) method (32); ^h^estimates derived using weighted mode estimator approach, where the largest group of instruments with consistent MR estimate are assumed to be valid (31); ^i^OR per standard deviation.

For genetically predicted CPD, we found little evidence for an association with overall breast cancer and ER subtypes using IVW random-effects method (Table 2, Supplementary Table 5 and Supplementary Figs. 2–4). There was no indication of horizontal pleiotropy based on the MR-Egger intercept test. Results across two-sample MR analyses were consistent in overall breast cancer and ER subtypes without any indication of horizontal pleiotropy from the MR-Egger intercept. The MR-PRESSO analysis detected three outlying SNPs (rs11940255, rs1737894 and rs73229090) although the outcome of IVW analysis after outlier removed remained unchanged in overall breast cancer and ER-positive tumour risk. No outlier was observed with ER-negative disease (Table 2, Supplementary Table 5 and Supplementary Figs. 2–4).

## Discussion

This MR study supports an association between genetically predicted lifetime smoking exposure and increasing invasive breast cancer risk but no clear association with cigarettes per day among smokers. The estimates based on the wPGS for the two smoking traits and several two-sample MR methods were consistent.

The LSI has not been assessed in studies of breast cancer risk but the modest association found in this analysis is in line with the modest associations of current and former smoking with invasive breast cancer risk reported in the recent large pooled analysis.^13^ We did not find support for an association between cigarettes per day in ever-smokers and invasive breast cancer risk, whereas modest associations were reported for cigarettes per day in current smokers compared with never-smokers in the pooled analysis. With the restriction to ever-smokers, the MR analysis of CPD had low statistical power for the very modest dose–response association estimated by our data (Table 1) as well as that reported in the pooled analysis.^13^

In addition to smoking exposure traits, we addressed two dichotomous smoking status traits, namely smoking initiation and smoking cessation, which account for the lifetime smoking exposure. Despite a pooled analysis of epidemiological studies reporting an increased risk of breast cancer associated with current smokers compared to non-smokers (OR of 1.02),^13^ a recent MR study found inconclusive evidence of association between genetically predicted smoking initiation and breast cancer risk using summary-level data (OR: 1.05, 95% CI: 0.99–1.12, *P* = 0.12 in BCAC; OR: 0.97, 95% CI: 0.90–1.06, *P* = 0.51 in UK Biobank).^39^ We conducted association analyses of wPGS of smoking initiation and smoking cessation with breast cancer risk (see Supplementary Note) and found no statically significant association for either smoking initiation (OR 1.05, 95% CI 0.99–1.11, *P* = 0.08) or smoking cessation (OR 1.06, 95% CI: 0.88–1.27, *P* = 0.52) (Supplementary Table 6). The results remained unchanged after adjusting for breast cancer risk factors (Supplementary Table 6). We cannot rule out that the MR analysis of the smoking status had low statistical power to detect the very modest association estimated by our data (Table 1) along with that reported in the pooled analysis.^13^ It is also possible that the MR analysis of smoking status especially smoking initiation alone does not capture the association between cigarette smoking and breast cancer risk comparing to the LSI which accounts for other smoking traits.

There are several not entirely resolved issues concerning the association between smoking and breast cancer risks, such as potential effect modification by timing of smoking exposure, menopausal status and oestrogen receptor (ER) status, potential confounding by alcohol consumption. We conducted an association analysis between wPGS of age at smoking initiation and invasive breast cancer (see Supplementary Note). The result showed an inverse association but statistically nonsignificant with low precision (OR 0.88, 95% CI: 0.25–3.07, *P* = 0.84) (Supplementary Table 6). This is in line with the result of a pooled analysis of epidemiological studies showing that women who started smoking later than 24 years old were at lower breast cancer risk than those who started smoking earlier when compared to non-smokers.^13^ Smoking initiation in relation to first birth has been considered an essential factor in the association with breast cancer risk since the undifferentiated breast epithelium is particularly susceptible to carcinogens before the first birth.^40^ Indeed this appears to be supported by findings of a stronger association with smoking with breast cancer risk if initiated before first birth and a stronger influence of smoking on breast cancer among women who started smoking more than 10 years before the first full-term pregnancy on breast cancer.^13,41–46^ Since the relevant information was only available for a subset of study participants, we did not have sufficient power to address potential differential associations according to timing of smoking exposure in relation to first birth.

Stronger associations between smoking and breast cancer among premenopausal women have been hypothesised since the morphology of the breast and the endogenous hormone levels change substantially during the menopausal transition, and menopausal status alter other breast cancer risk factors.^10^ The MR results confirmed the lack of effect modification by menopausal status also reported by previous epidemiological studies.^13,47,48^ Despite early evidence against a differential association by ER status,^2,3^ recent epidemiologic studies reported a stronger association for risk of ER-positive breast cancer.^10,13,41^ We did not find clear evidence for heterogeneity by ER status although power to detect effect heterogeneity was limited particularly due to the small sample size for ER-negative disease.

Previous epidemiologic studies have addressed the confounding effect by alcohol consumption and found an association of smoking with breast cancer risk after stratifying on alcohol consumption.^13,41–43^ We addressed this issue by excluding SNPs associated with alcohol intake from the wPGS. However, the results did not change significantly in sensitivity analyses with wPGS, including the overlapping alcohol consumption-associated SNPs (Supplementary Table 7). Also, our multivariable analyses adjusting for alcohol consumption yielded association estimates that remained unchanged. The lower precision can be attributed to the reduced dataset that required information on alcohol intake with the ensuing diminished power.

Despite epidemiological evidence indicating alcohol consumption as an established risk factor for breast cancer risk,^49^ a recent MR study reported no significant association of breast cancer risk with genetically predicted alcohol intake using summary association data.^39^ We conducted an analysis between wPGS of alcohol consumption and invasive breast cancer using individual-level data (see Supplementary Note). We found no clear evidence of association between genetically predicted alcohol consumption and invasive breast cancer (OR: 0.98, 95% CI: 0.86–1.11, *P* = 0.74). The association remained unchanged after adjusting for LSI (OR: 0.88, 95% CI: 0.72–1.09, *P* = 0.25).

### Strengths and limitations

Despite the relatively large sample size used for our analyses, weak statistical power to detect modest associations should be considered when interpreting the results. The power calculation shows that our study had 68% power to detect an OR of 1.20 per SD change in LSI but only around 9% power for an OR of 1.05 in combined dataset (iCOGS and OncoArray) (Supplementary Table 3). Since the reported order of magnitude for associations with CPD is around 1.05 per ten cigarettes,^13^ we cannot exclude that the lack of association observed in the MR analysis particularly regarding CPD may be due to limited power. The unit for LSI is not scaled due to the nature of the phenotype. The reported order of magnitude for associations with LSI can therefore not be directly compared with the relative risk estimates for smoking from observational studies.

MR estimates have a causal interpretation only if the assumptions of the instrumental variable approach hold. Even though we performed extensive sensitivity analyses to detect potential violations, it is difficult to prove the validity of the assumptions. The LSI captures multiple aspects of smoking behaviours, which could have introduced more potential for horizontal pleiotropy. The more diffuse the definition of smoking, the more lifestyle factors might be correlated, making it especially important to test for horizontal pleiotropy. No evidence of pleiotropic effects was found by conducting various sensitivity analyses; however, residual pleiotropy is difficult to exclude and should be considered.

The genetic instrument for LSI allows for the use of the large entire sample to conduct MR analysis without stratifying on smoking status. The analyses for CPD were restricted to smokers by reason that the CPD-associated SNPs were identified among ever-smokers in the GWAS study,^21^ which reduce statistical power to detect an association. Moreover, we should note that restricting to ever-smokers may induce a sampling bias and invalidate the MR assumptions. By restricting to smokers, smoking initiation can open up the path from exposure (wPGS for CPD) to outcome (breast cancer risk). It can make the association between smoking and breast cancer risk appear weaker by removing a part of the association that is attributable to smoking initiation.

Another limitation is that our analysis was restricted to participants of European ancestry; therefore, our results may not apply to populations of other ethnicities. However, it reduces the potential bias caused by population stratification.

### Conclusion

In conclusion, this Mendelian randomisation analysis using both individual-level data and summary statistics supports a causal association between lifetime smoking exposure and breast cancer risk. Larger studies for MR analysis are warranted to address additional aspects of smoking behaviour.

## Supplementary information

Supplementary Materials

## Data Availability

Availability of lifetime smoking index GWAS described by Wootton et al.^20^ Availability of self-reported cigarettes per day GWAS described by Liu et al.^21^ Individual genotyping data from BCAC will not be made publicly available due to restraints imposed by the ethics committees of individual studies; requests for data can be made to the Data Access Coordination Committee of BCAC (http://bcac.ccge.medschl.cam.ac.uk/). Summary results for all variants genotyped by BCAC are available at http://bcac.ccge.medschl.cam.ac.uk/.
