# Supplementary material for: Mendelian randomisation study of smoking exposure in relation to breast cancer risk
Source: Br J Cancer. 2021 Aug 2;125(8):1135–45. doi: 10.1038/s41416-021-01432-8 (PMC8505411; doi:10.1038/s41416-021-01432-8)
Supplement: Supplementary file 1 — Supplementary Materials [file 41416_2021_1432_MOESM1_ESM.docx]

# **Supplementary Materials**

## **Table S1. Selected characteristics of participants in the BCAC iCOGS and OncoArray projects.**

|  |  | **iCOGS** | |  | **OncoArray** | |
| --- | --- | --- | --- | --- | --- | --- |
| **Variables** |  | **Case** | **Control** |  | **Case** | **Control** |
| Age at interview/diagosis (years), mean ± SD, N=196,101 |  | 55.50 ± 12.53 | 56.36 ± 12.52 |  | 56.39 ± 12.32 | 55.94 ± 12.00 |
| Age at menarche (years), mean ± SD, N=138,752 |  | 12.86 ± 1.53 | 13.02 ± 1.52 |  | 12.96 ± 1.56 | 12.94 ± 1.55 |
| Age at first live birth (years), mean ± SD, N=133,915 |  | 21.22 ± 10.18 | 21.71 ± 9.68 |  | 21.00 ± 10.38 | 21.41 ± 9.49 |
| Age at menopause (years), mean ± SD, N=76,292 |  | 47.80 ± 6.21 | 48.29 ± 5.99 |  | 48.40 ± 6.02 | 48.08 ± 6.13 |
| BMI (kg/m2), mean ± SD, N=143,245 |  | 26.10 ± 5.10 | 25.97 ± 4.97 |  | 26.32 ± 5.17 | 26.15 ± 5.10 |
| Ever breastfeeding ^a^, N (%) |  | 13,639 (33.8) | 10,878 (28.4) |  | 20,642 (30.2) | 17,071 (31.8) |
| Ever use of MTH (postmenopausal) ^b^, N (%) |  | 7,378 (18.3) | 6,759 (17.6) |  | 17,165 (25.1) | 16,173 (30.1) |
| Postmenopausal status ^c^, N (%) |  | 18,003 (44.6) | 18,912 (49.4) |  | 33,795 (49.5) | 28,925 (53.8) |
| Family history of breast cancer (yes) ^d^, N (%) |  | 7,616 (18.9) | 2,636 (6.9) |  | 13,910 (20.4) | 6,134 (11.4) |
| Parous (yes) ^e^, N (%) |  | 23,370 (57.8) | 23,887 (62.3) |  | 43,289 (63.4) | 39,133 (72.8) |
| Frequency alcoholic drinks (glasses / day), mean ± SD, N=45,131 |  | 0.48 ± 1.03 | 0.39 ± 0.85 |  | 0.54 ± 1.08 | 0.54 ± 0.90 |
| Parity (numbers), mean ± SD, N=149,539 |  | 2.09 ± 1.41 | 2.14 ± 1.32 |  | 1.98 ± 1.32 | 2.19 ± 1.36 |
| Education level ^f^, N (%)  Primary education  Secondary education  Tertiary education |  | 2,357 (5.8)  3,900 (9.6)  2,738 (6.8) | 1,662 (4.3)  6,127 (16.0)  3,587 (9.4) |  | 5,867 (8.6)  17,168 (25.1)  11,681 (17.1) | 4,078 (7.6)  17,703 (32.9)  13,177 (24.5) |
| Cigarette smoking status ^g^, N (%)  Never smokers  Former smokers  Current smokers |  | 6,733 (16.7)  4,382 (10.8)  2,960 (7.3) | 9,641 (25.2)  6,044 (15.8)  2,446 (6.4) |  | 21,779 (31.9)  12,531 (18.3)  6,283 (9.2) | 21,682 (40.4)  12,320 (22.9)  5,383 (10.0) |
| Total duration of smoking (years), mean ± SD, N=78,252 |  | 9.43 ± 15.33 | 8.37 ± 13.36 |  | 9.25±14.81 | 8.60 ± 13.40 |
| Cigarettes per day (pack of cigarettes per day), mean ± SD, N=77,010 |  | 1.27 ± 0.44 | 0.22 ± 0.42 |  | 0.27 ± 0.45 | 0.26 ± 0.45 |
| Age at starting regular smoking (years), mean ± SD, N=31,366 |  | 19.13 ± 5.46 | 20.27 ± 6.67 |  | 19.60 ± 5.92 | 19.73 ± 5.78 |
| Age at stopping regular smoking (years), mean ± SD, N=21,467 |  | 38.04 ± 12.82 | 39.59 ± 12.54 |  | 38.91 ± 12.71 | 38.68 ± 12.54 |
| Lifetime smoking index ^h^, mean ± SD, N= 85,374 |  | 0.64 ± 2.51 | 0.52 ± 2.21 |  | 0.60 ± 2.41 | 0.57 ± 2.27 |
| Smoking pack-years (pack-years) ^i^, mean ± SD, N=87,365 |  | 6.59 ± 13.81 | 5.69 ± 12.47 |  | 6.69 ± 14.74 | 6.11 ± 12.24 |

^a^ The numbers of missing information were 19,503 (48.3%) in iCOGS cases, 20,766 (54.2%) in iCOGS controls, 33,416 (48.9%) in OncoArray cases, and 26,159 (48.7%) in OncoArray controls; ^b^ The numbers of missing information were 22,579 (55.9%) in iCOGS cases, 20,813 (54.3%) in iCOGS controls, 23,582 (34.5%) in OncoArray cases, and 14,656 (27.3%) in OncoArray controls; ^c^ The numbers of missing information were 13,150 (32.6%) in iCOGS cases, 10,783 (28.1%) in iCOGS controls, 19,409 (28.4%) in OncoArray cases, and 13,597 (25.3%) in OncoArray controls; ^d^ The numbers of missing information were 11,423 (28.3%) in iCOGS cases, 16,282 (42.5%) in iCOGS controls, 18,869 (27.6%) in OncoArray cases, and 18,654 (34.7%) in OncoArray controls; ^e^ The numbers of missing information were 12,707 (31.5%) in iCOGS cases, 10,270 (26.8%) in iCOGS controls, 17,178 (25.1%) in OncoArray cases, and 9,141 (17.0%) in OncoArray controls; ^f^ The numbers of missing information were 31,404 (77.7%) in iCOGS cases, 26,938 (70.3%) in iCOGS controls, 33,603 (49.2%) in OncoArray cases, and 18,763 (34.9%) in OncoArray controls; ^g^ The numbers of missing information were 26,324 (65.2%) in iCOGS cases, 20,183 (52.7%) in iCOGS controls, 27,726 (40.6%) in OncoArray cases, and 14,336 (26.7%) in OncoArray controls; ^h^ Lifetime smoking index = (1 – 0.5^(*dur*/*τ*)) (0.5^(*tsc*/*τ*)) ln(*int*+1) (*dur*: duration of smoking; *int*: cigarettes per day; *τ:* half-life capturing the exponentially decreasing effect of smoking at a given time on health outcomes; *tsc*: time since smoking cessation). The value of half-life and lag-time was determined by simulating the effects of lifetime smoking on breast cancer in the BCAC studies: suggested the best fitting value as 15 for half-life and 0 for lag-time. In our data, LSI values for ever smokers ranged from 0.0027 (an individual who smoked 1 cigarette per day for 1 year) to 3.77 (a 71 years old individual who currently smokes 60 a day and started smoking at age 17) and for never-smokers LSI was coded 0. Full details on the construction of the lifetime smoking index can be found in Supplementary Note of Wootton, Richmond et al. (2019)^1^. ^i^ Pack-year was calculated as: (average number of cigarettes per day)*(number of years smoked)/20.

Abbreviation: MHT, menopausal hormone therapy; SD, standard deviation.

## **Table S2. Associations between individual SNPs in each genetic instrument and breast cancer risk in the BCAC.**

| **SNP** | **Chr** | **Position (hg19)** | **EA** | **OA** | **Trait** | **EAF ^a^** |  | **Overall BC** | |  | **ER positive** | |  | **ER negative** | | **LD with AC-associated SNP ^b^** |
| --- | --- | --- | --- | --- | --- | --- | --- | --- | --- | --- | --- | --- | --- | --- | --- | --- |
|  |  |  |  |  |  |  |  | **β** | **SE** |  | **β** | **SE** |  | **β** | **SE** |  |
| rs10918701 | 1 | 162090536 | A | G | LSI | 0.63 |  | -6.2E-03 | 6.5E-03 |  | -5.7E-03 | 7.7E-03 |  | -2.1E-02 | 1.2E-02 |  |
| rs10922907 | 1 | 91193049 | T | A | LSI | 0.54 |  | 8.9E-03 | 6.8E-03 |  | 4.8E-03 | 8.1E-03 |  | 8.1E-03 | 1.2E-02 |  |
| rs11210229 | 1 | 73860028 | G | A | LSI | 0.60 |  | -2.6E-03 | 6.4E-03 |  | -1.3E-03 | 7.6E-03 |  | -2.3E-02 | 1.2E-02 |  |
| rs1193237 | 1 | 7526486 | C | G | LSI | 0.56 |  | -7.4E-03 | 6.9E-03 |  | -8.9E-03 | 8.2E-03 |  | 4.5E-03 | 1.2E-02 |  |
| rs1931263 | 1 | 96175101 | T | G | LSI | 0.49 |  | 1.2E-02 | 6.2E-03 |  | 5.8E-03 | 7.4E-03 |  | 1.6E-02 | 1.1E-02 |  |
| rs1933270 | 1 | 49977965 | G | T | LSI | 0.63 |  | 6.7E-03 | 6.3E-03 |  | 1.2E-02 | 7.6E-03 |  | 1.7E-02 | 1.2E-02 |  |
| rs4949465 | 1 | 32178489 | C | T | LSI | 0.14 |  | -4.3E-03 | 9.8E-03 |  | -6.5E-03 | 1.2E-02 |  | 1.1E-02 | 1.8E-02 |  |
| rs549845 | 1 | 44076469 | A | G | LSI | 0.70 |  | 1.4E-02 | 7.0E-03 |  | 2.1E-02 | 8.3E-03 |  | 2.0E-04 | 1.3E-02 |  |
| rs7519626 | 1 | 99514554 | T | C | LSI | 0.66 |  | -2.3E-03 | 6.7E-03 |  | -2.3E-03 | 8.0E-03 |  | -1.1E-02 | 1.2E-02 |  |
| rs7528604 | 1 | 66407352 | A | G | LSI | 0.43 |  | 1.7E-02 | 6.5E-03 |  | 2.6E-02 | 7.8E-03 |  | 1.1E-02 | 1.2E-02 | rs12088813, r^2^=0.54 |
| rs7553348 | 1 | 75005067 | A | G | LSI | 0.56 |  | 9.2E-03 | 6.2E-03 |  | 1.2E-02 | 7.4E-03 |  | 1.3E-02 | 1.1E-02 |  |
| rs9435340 | 1 | 107593201 | A | T | LSI | 0.67 |  | -1.3E-03 | 6.6E-03 |  | -5.0E-04 | 7.8E-03 |  | -1.7E-02 | 1.2E-02 |  |
| rs11264100 | 1 | 35591626 | G | A | CPD | 0.89 |  | -3.0E-02 | 1.0E-02 |  | -3.9E-02 | 1.2E-02 |  | -2.9E-03 | 1.9E-02 |  |
| rs2072659 | 1 | 154548521 | G | C | CPD | 0.11 |  | 3.2E-03 | 1.1E-02 |  | -3.5E-03 | 1.3E-02 |  | 4.3E-03 | 2.0E-02 |  |
| rs34973462 | 1 | 175993820 | T | C | CPD | 0.35 |  | 1.0E-02 | 6.5E-03 |  | 1.8E-02 | 7.8E-03 |  | -1.5E-03 | 1.2E-02 |  |
| rs12623702 | 2 | 202885506 | G | A | LSI | 0.39 |  | -7.9E-03 | 6.5E-03 |  | -1.5E-02 | 7.7E-03 |  | 1.9E-02 | 1.2E-02 |  |
| rs13009008 | 2 | 174043233 | G | A | LSI | 0.67 |  | -5.5E-03 | 6.6E-03 |  | -6.3E-03 | 7.8E-03 |  | -8.2E-03 | 1.2E-02 |  |
| rs13016665 | 2 | 57995348 | A | C | LSI | 0.44 |  | -1.6E-02 | 6.3E-03 |  | -1.9E-02 | 7.6E-03 |  | -1.1E-02 | 1.2E-02 |  |
| rs2678670 | 2 | 104469564 | T | A | LSI | 0.52 |  | -1.3E-02 | 6.2E-03 |  | -1.4E-02 | 7.4E-03 |  | -1.5E-02 | 1.1E-02 |  |
| rs2867112 | 2 | 651349 | G | T | LSI | 0.17 |  | 3.7E-02 | 8.1E-03 |  | 2.7E-02 | 9.7E-03 |  | 7.0E-02 | 1.5E-02 |  |
| rs2890772 | 2 | 146175106 | T | G | LSI | 0.59 |  | -2.3E-03 | 6.4E-03 |  | 8.0E-04 | 7.6E-03 |  | -1.7E-02 | 1.2E-02 |  |
| rs359243 | 2 | 60475509 | C | T | LSI | 0.60 |  | 6.9E-03 | 6.4E-03 |  | 1.3E-02 | 7.6E-03 |  | -2.1E-02 | 1.2E-02 |  |
| rs3769949 | 2 | 166199284 | A | T | LSI | 0.46 |  | 3.4E-03 | 6.4E-03 |  | -5.3E-03 | 7.6E-03 |  | 1.1E-02 | 1.2E-02 |  |
| rs3811038 | 2 | 113240183 | C | T | LSI | 0.28 |  | 5.5E-03 | 6.9E-03 |  | 1.5E-02 | 8.2E-03 |  | -2.0E-02 | 1.3E-02 |  |
| rs4473348 | 2 | 182073742 | T | A | LSI | 0.76 |  | -3.0E-03 | 7.3E-03 |  | -8.0E-04 | 8.7E-03 |  | 9.5E-03 | 1.3E-02 |  |
| rs4671357 | 2 | 60136176 | C | T | LSI | 0.49 |  | 5.5E-03 | 6.4E-03 |  | 5.3E-03 | 7.6E-03 |  | 6.6E-03 | 1.2E-02 |  |
| rs62135536 | 2 | 44326028 | T | C | LSI | 0.03 |  | -1.7E-02 | 2.0E-02 |  | -1.5E-02 | 2.4E-02 |  | -9.1E-03 | 3.7E-02 |  |
| rs62155874 | 2 | 105973094 | G | A | LSI | 0.12 |  | -9.1E-03 | 9.6E-03 |  | -5.8E-03 | 1.1E-02 |  | -4.8E-03 | 1.8E-02 |  |
| rs62175972 | 2 | 161362830 | C | T | LSI | 0.04 |  | -2.4E-02 | 1.7E-02 |  | -2.9E-02 | 2.0E-02 |  | -4.0E-02 | 3.1E-02 |  |
| rs6741228 | 2 | 22548774 | C | T | LSI | 0.56 |  | -1.1E-03 | 6.3E-03 |  | 9.0E-04 | 7.5E-03 |  | -7.7E-03 | 1.2E-02 |  |
| rs7569203 | 2 | 45154418 | C | A | LSI | 0.33 |  | -4.3E-03 | 6.6E-03 |  | -1.1E-02 | 7.9E-03 |  | 8.3E-03 | 1.2E-02 | rs13383034, r^2^=1.0 |
| rs10204824 | 2 | 148372720 | G | A | CPD | 0.65 |  | -1.1E-02 | 6.9E-03 |  | -9.8E-03 | 8.2E-03 |  | -4.4E-03 | 1.3E-02 |  |
| rs7599488 | 2 | 60718347 | T | C | CPD | 0.43 |  | 1.5E-02 | 6.3E-03 |  | 1.4E-02 | 7.6E-03 |  | 1.6E-02 | 1.2E-02 |  |
| rs78408772 | 2 | 62710608 | T | C | CPD | 0.10 |  | 1.6E-02 | 1.0E-02 |  | 2.2E-02 | 1.2E-02 |  | -5.6E-03 | 1.9E-02 |  |
| rs326341 | 3 | 107811142 | A | G | LSI | 0.46 |  | -4.0E-04 | 6.4E-03 |  | 8.0E-03 | 7.6E-03 |  | -3.8E-03 | 1.2E-02 |  |
| rs421983 | 3 | 84892866 | C | T | LSI | 0.49 |  | -2.5E-03 | 6.4E-03 |  | 1.3E-03 | 7.6E-03 |  | 5.0E-04 | 1.2E-02 |  |
| rs6778080 | 3 | 49317338 | C | T | LSI | 0.73 |  | -1.5E-02 | 7.0E-03 |  | -1.4E-02 | 8.4E-03 |  | -1.8E-02 | 1.3E-02 |  |
| rs6779302 | 3 | 16859710 | T | G | LSI | 0.37 |  | -2.4E-03 | 6.4E-03 |  | 5.3E-03 | 7.7E-03 |  | -4.6E-03 | 1.2E-02 |  |
| rs73220544 | 3 | 131074511 | C | A | LSI | 0.17 |  | 4.9E-03 | 9.0E-03 |  | 7.3E-03 | 1.1E-02 |  | 5.0E-04 | 1.6E-02 |  |
| rs775758 | 3 | 77582005 | T | A | LSI | 0.56 |  | 1.5E-03 | 6.8E-03 |  | -5.5E-03 | 8.1E-03 |  | -5.0E-03 | 1.2E-02 |  |
| rs9842947 | 3 | 157412246 | T | C | LSI | 0.69 |  | -1.2E-03 | 6.9E-03 |  | 3.2E-03 | 8.2E-03 |  | 8.0E-03 | 1.3E-02 |  |
| rs2084533 | 3 | 16872929 | T | C | CPD | 0.32 |  | -3.2E-03 | 6.7E-03 |  | 2.8E-03 | 8.0E-03 |  | 2.5E-03 | 1.2E-02 |  |
| rs2236951 | 3 | 50421081 | C | T | CPD | 0.20 |  | 1.8E-02 | 7.8E-03 |  | 2.3E-02 | 9.4E-03 |  | 3.6E-03 | 1.4E-02 |  |
| rs28813180 | 3 | 158083918 | A | G | CPD | 0.50 |  | -1.6E-02 | 6.2E-03 |  | -1.7E-02 | 7.4E-03 |  | -1.3E-02 | 1.1E-02 | rs6787172, r^2^=0.59 |
| rs699165 | 3 | 136224697 | G | A | CPD | 0.76 |  | -1.1E-02 | 7.3E-03 |  | -4.8E-03 | 8.7E-03 |  | -3.0E-02 | 1.3E-02 |  |
| rs7431710 | 3 | 48935583 | A | G | CPD | 0.64 |  | 1.9E-03 | 6.5E-03 |  | 1.7E-03 | 7.8E-03 |  | 1.9E-02 | 1.2E-02 |  |
| rs17576594 | 4 | 147952241 | A | G | LSI | 0.28 |  | -1.8E-03 | 6.9E-03 |  | 7.7E-03 | 8.2E-03 |  | 3.0E-04 | 1.3E-02 |  |
| rs317021 | 4 | 35418368 | A | T | LSI | 0.17 |  | 9.3E-03 | 9.1E-03 |  | 6.8E-03 | 1.1E-02 |  | 2.0E-02 | 1.7E-02 |  |
| rs61796681 | 4 | 23678196 | T | A | LSI | 0.08 |  | 8.2E-03 | 1.1E-02 |  | 8.1E-03 | 1.4E-02 |  | -7.3E-03 | 2.1E-02 |  |
| rs624833 | 4 | 2881256 | G | T | LSI | 0.31 |  | -1.3E-03 | 6.7E-03 |  | 7.5E-03 | 8.0E-03 |  | -1.3E-02 | 1.2E-02 |  |
| rs72678864 | 4 | 112422145 | A | G | LSI | 0.15 |  | 6.1E-03 | 9.0E-03 |  | 4.5E-03 | 1.1E-02 |  | 6.6E-03 | 1.6E-02 |  |
| rs1024323 | 4 | 3006043 | T | C | CPD | 0.39 |  | 5.0E-03 | 6.5E-03 |  | 1.2E-02 | 7.7E-03 |  | 6.9E-03 | 1.2E-02 |  |
| rs10454798 | 4 | 67980830 | T | G | CPD | 0.27 |  | -1.9E-02 | 7.1E-03 |  | -2.4E-02 | 8.4E-03 |  | -2.1E-02 | 1.3E-02 |  |
| rs11940255 | 4 | 67086288 | A | G | CPD | 0.71 |  | 2.4E-02 | 7.5E-03 |  | 2.9E-02 | 9.0E-03 |  | 1.3E-02 | 1.4E-02 |  |
| rs10052591 | 5 | 50812738 | C | T | LSI | 0.43 |  | 2.8E-03 | 6.3E-03 |  | -3.6E-03 | 7.5E-03 |  | 2.0E-02 | 1.1E-02 |  |
| rs11948770 | 5 | 13246336 | C | T | LSI | 0.24 |  | 4.7E-03 | 7.3E-03 |  | 4.7E-03 | 8.7E-03 |  | 1.0E-02 | 1.3E-02 |  |
| rs13153393 | 5 | 167604213 | G | A | LSI | 0.11 |  | 1.3E-02 | 1.1E-02 |  | 1.6E-02 | 1.3E-02 |  | -1.5E-02 | 1.9E-02 |  |
| rs2080870 | 5 | 60388313 | T | A | LSI | 0.75 |  | 1.5E-02 | 7.1E-03 |  | 1.8E-02 | 8.5E-03 |  | -9.6E-03 | 1.3E-02 |  |
| rs245774 | 5 | 170530930 | G | A | LSI | 0.72 |  | 4.0E-04 | 6.9E-03 |  | 3.4E-03 | 8.3E-03 |  | -4.9E-03 | 1.3E-02 |  |
| rs329120 | 5 | 133861756 | T | C | LSI | 0.42 |  | -2.1E-03 | 6.3E-03 |  | -3.3E-03 | 7.5E-03 |  | 1.3E-02 | 1.1E-02 |  |
| rs4571506 | 5 | 87756918 | T | C | LSI | 0.47 |  | 7.2E-03 | 6.2E-03 |  | -2.0E-03 | 7.4E-03 |  | 1.2E-02 | 1.1E-02 | rs4916723, r^2^=0.47 |
| rs4957528 | 5 | 106420589 | C | A | LSI | 0.79 |  | 2.1E-02 | 8.1E-03 |  | 2.6E-02 | 9.7E-03 |  | 2.7E-03 | 1.5E-02 |  |
| rs71627581 | 5 | 43161351 | A | G | LSI | 0.11 |  | -9.8E-03 | 1.1E-02 |  | -2.0E-03 | 1.3E-02 |  | 1.3E-02 | 1.9E-02 |  |
| rs986391 | 5 | 166993972 | A | G | LSI | 0.63 |  | -1.2E-02 | 7.2E-03 |  | -2.1E-02 | 8.6E-03 |  | -3.6E-03 | 1.3E-02 |  |
| rs12202536 | 6 | 67475273 | G | A | LSI | 0.48 |  | 1.0E-03 | 6.2E-03 |  | -1.0E-04 | 7.4E-03 |  | 3.0E-03 | 1.1E-02 |  |
| rs2254710 | 6 | 37477000 | A | C | LSI | 0.76 |  | 5.0E-04 | 7.3E-03 |  | 1.8E-03 | 8.7E-03 |  | -1.5E-03 | 1.3E-02 |  |
| rs2894808 | 6 | 52861990 | A | T | LSI | 0.08 |  | 1.8E-02 | 1.2E-02 |  | 9.4E-03 | 1.4E-02 |  | 3.5E-02 | 2.1E-02 |  |
| rs6935954 | 6 | 26255451 | G | A | LSI | 0.59 |  | 4.7E-03 | 6.4E-03 |  | 3.0E-04 | 7.7E-03 |  | 9.8E-03 | 1.2E-02 |  |
| rs7766610 | 6 | 111707821 | A | C | LSI | 0.81 |  | 4.7E-03 | 8.0E-03 |  | 1.3E-02 | 9.5E-03 |  | 1.6E-02 | 1.5E-02 |  |
| rs7766641 | 6 | 26184102 | A | G | CPD | 0.29 |  | 1.6E-02 | 6.9E-03 |  | 1.8E-02 | 8.2E-03 |  | 1.1E-02 | 1.3E-02 |  |
| rs10226228 | 7 | 32315613 | G | A | LSI | 0.36 |  | -1.2E-02 | 6.5E-03 |  | -1.3E-02 | 7.8E-03 |  | -1.1E-02 | 1.2E-02 |  |
| rs10282292 | 7 | 111092478 | T | C | LSI | 0.64 |  | 6.1E-03 | 6.7E-03 |  | 9.5E-03 | 8.0E-03 |  | -1.3E-02 | 1.2E-02 |  |
| rs11768481 | 7 | 96629103 | A | C | LSI | 0.35 |  | -7.0E-03 | 7.7E-03 |  | -4.2E-03 | 9.2E-03 |  | -1.6E-02 | 1.4E-02 |  |
| rs1922018 | 7 | 3560401 | T | C | LSI | 0.64 |  | -2.4E-03 | 6.5E-03 |  | -5.0E-04 | 7.8E-03 |  | -2.0E-02 | 1.2E-02 |  |
| rs2401924 | 7 | 115057862 | C | G | LSI | 0.49 |  | -3.1E-03 | 6.5E-03 |  | -4.9E-03 | 7.8E-03 |  | 4.3E-03 | 1.2E-02 |  |
| rs4731925 | 7 | 132664757 | T | C | LSI | 0.69 |  | -9.1E-03 | 6.9E-03 |  | -7.7E-03 | 8.3E-03 |  | -1.6E-02 | 1.3E-02 |  |
| rs6957896 | 7 | 132309592 | T | C | LSI | 0.48 |  | -4.3E-03 | 6.8E-03 |  | -3.7E-03 | 8.1E-03 |  | -1.4E-02 | 1.2E-02 |  |
| rs6962772 | 7 | 99081730 | G | A | LSI | 0.16 |  | -7.1E-03 | 8.5E-03 |  | 2.7E-03 | 1.0E-02 |  | -1.1E-02 | 1.6E-02 | rs10236149, r^2^=0.74 |
| rs7807019 | 7 | 117543063 | G | A | LSI | 0.49 |  | 0.0E+00 | 6.3E-03 |  | 7.2E-03 | 7.5E-03 |  | -1.5E-02 | 1.1E-02 |  |
| rs215600 | 7 | 32333642 | A | G | CPD | 0.65 |  | 1.3E-02 | 6.6E-03 |  | 1.3E-02 | 7.9E-03 |  | 1.1E-02 | 1.2E-02 |  |
| rs62447179 | 7 | 50339609 | A | G | CPD | 0.30 |  | -6.0E-03 | 7.6E-03 |  | -6.6E-03 | 9.1E-03 |  | 4.0E-04 | 1.4E-02 |  |
| rs11783093 | 8 | 27425349 | T | C | LSI | 0.15 |  | -2.9E-02 | 8.8E-03 |  | -4.4E-02 | 1.1E-02 |  | 1.2E-02 | 1.6E-02 |  |
| rs2062882 | 8 | 91839576 | A | G | LSI | 0.42 |  | 5.8E-03 | 6.5E-03 |  | 7.0E-04 | 7.8E-03 |  | 1.7E-02 | 1.2E-02 |  |
| rs35169606 | 8 | 9604066 | G | T | LSI | 0.39 |  | 3.5E-03 | 7.3E-03 |  | -3.7E-03 | 8.8E-03 |  | 2.2E-02 | 1.3E-02 |  |
| rs72674867 | 8 | 95578201 | T | A | LSI | 0.26 |  | 3.2E-03 | 7.1E-03 |  | 5.1E-03 | 8.5E-03 |  | -3.7E-03 | 1.3E-02 |  |
| rs13253502 | 8 | 42442018 | A | G | CPD | 0.40 |  | 3.9E-03 | 7.1E-03 |  | 5.8E-03 | 8.5E-03 |  | 7.3E-03 | 1.3E-02 |  |
| rs2741351 | 8 | 27418040 | C | A | CPD | 0.82 |  | -2.5E-03 | 8.2E-03 |  | -2.0E-03 | 9.8E-03 |  | 1.6E-02 | 1.5E-02 |  |
| rs4236926 | 8 | 42578059 | G | T | CPD | 0.77 |  | 1.5E-02 | 7.4E-03 |  | 9.5E-03 | 8.8E-03 |  | 2.0E-02 | 1.4E-02 |  |
| rs73229090 | 8 | 27442127 | A | C | CPD | 0.11 |  | -3.1E-02 | 1.0E-02 |  | -3.8E-02 | 1.2E-02 |  | 7.0E-03 | 1.9E-02 |  |
| rs790564 | 8 | 64604218 | C | A | CPD | 0.73 |  | 4.2E-03 | 7.6E-03 |  | -1.0E-03 | 9.0E-03 |  | 5.5E-03 | 1.4E-02 | rs1217091, r^2^=0.62 |
| rs113382419 | 9 | 136463019 | A | C | LSI | 0.10 |  | 1.5E-02 | 1.1E-02 |  | 1.6E-02 | 1.3E-02 |  | 8.9E-03 | 1.9E-02 |  |
| rs1221148 | 9 | 122046875 | G | C | LSI | 0.41 |  | -1.2E-02 | 6.9E-03 |  | -5.9E-03 | 8.3E-03 |  | -2.0E-02 | 1.3E-02 |  |
| rs1246265 | 9 | 86761745 | C | T | LSI | 0.70 |  | 2.4E-03 | 6.7E-03 |  | 9.0E-04 | 8.0E-03 |  | 1.4E-02 | 1.2E-02 |  |
| rs13296519 | 9 | 128471924 | T | G | LSI | 0.39 |  | -2.9E-03 | 6.7E-03 |  | -5.9E-03 | 8.0E-03 |  | 6.6E-03 | 1.2E-02 |  |
| rs4543592 | 9 | 3014254 | C | T | LSI | 0.47 |  | -4.1E-03 | 6.3E-03 |  | 1.7E-03 | 7.5E-03 |  | 5.7E-03 | 1.1E-02 |  |
| rs7039819 | 9 | 82430418 | A | G | LSI | 0.59 |  | -8.1E-03 | 6.7E-03 |  | -1.1E-03 | 8.0E-03 |  | -1.6E-02 | 1.2E-02 |  |
| rs3025383 | 9 | 136502369 | C | T | CPD | 0.19 |  | -1.2E-03 | 8.1E-03 |  | -1.9E-03 | 9.7E-03 |  | 9.7E-03 | 1.5E-02 |  |
| rs75596189 | 9 | 136468701 | T | C | CPD | 0.10 |  | 1.6E-02 | 1.1E-02 |  | 1.7E-02 | 1.3E-02 |  | 1.0E-02 | 1.9E-02 |  |
| rs10823968 | 10 | 74738269 | T | A | LSI | 0.38 |  | -5.0E-03 | 6.5E-03 |  | -3.3E-03 | 7.7E-03 |  | -4.4E-03 | 1.2E-02 |  |
| rs11255908 | 10 | 8802912 | G | T | LSI | 0.25 |  | 2.2E-02 | 7.4E-03 |  | 2.6E-02 | 8.8E-03 |  | 2.7E-03 | 1.3E-02 |  |
| rs12244388 | 10 | 104640052 | A | G | LSI | 0.36 |  | 1.4E-02 | 6.5E-03 |  | 1.6E-02 | 7.7E-03 |  | 3.7E-03 | 1.2E-02 |  |
| rs17553262 | 10 | 92912773 | C | A | LSI | 0.11 |  | 1.0E-02 | 1.0E-02 |  | 7.3E-03 | 1.2E-02 |  | 2.1E-02 | 1.8E-02 |  |
| rs2675638 | 10 | 63576286 | A | G | LSI | 0.41 |  | -6.9E-03 | 6.4E-03 |  | -7.6E-03 | 7.6E-03 |  | -2.2E-02 | 1.2E-02 |  |
| rs3896224 | 10 | 106467853 | G | A | LSI | 0.43 |  | -1.5E-02 | 6.2E-03 |  | -9.6E-03 | 7.5E-03 |  | -1.9E-02 | 1.1E-02 |  |
| rs7077678 | 10 | 104438565 | T | C | LSI | 0.39 |  | -6.9E-03 | 6.4E-03 |  | -1.2E-02 | 7.6E-03 |  | -4.0E-04 | 1.2E-02 |  |
| rs112282219 | 11 | 46632809 | A | G | LSI | 0.04 |  | 4.3E-02 | 1.7E-02 |  | 5.8E-02 | 2.0E-02 |  | 1.9E-02 | 3.1E-02 |  |
| rs17309874 | 11 | 27667236 | A | G | LSI | 0.25 |  | -1.1E-02 | 7.2E-03 |  | -1.7E-02 | 8.6E-03 |  | -1.4E-03 | 1.3E-02 |  |
| rs34866095 | 11 | 16377356 | G | A | LSI | 0.31 |  | -1.6E-03 | 6.7E-03 |  | -6.6E-03 | 8.0E-03 |  | -2.0E-03 | 1.2E-02 |  |
| rs4391802 | 11 | 28674592 | G | A | LSI | 0.30 |  | 2.6E-03 | 7.2E-03 |  | 6.3E-03 | 8.6E-03 |  | 8.6E-03 | 1.3E-02 |  |
| rs75742406 | 11 | 17070365 | A | G | LSI | 0.26 |  | 1.7E-03 | 7.4E-03 |  | -1.7E-03 | 8.8E-03 |  | 1.4E-02 | 1.3E-02 |  |
| rs9919670 | 11 | 112877304 | A | G | LSI | 0.41 |  | -4.0E-03 | 6.3E-03 |  | 3.0E-04 | 7.6E-03 |  | -8.6E-03 | 1.2E-02 |  |
| rs10742683 | 11 | 43667625 | A | G | CPD | 0.42 |  | -3.2E-03 | 6.3E-03 |  | -1.8E-03 | 7.5E-03 |  | 4.0E-03 | 1.2E-02 |  |
| rs113001570 | 11 | 46737412 | T | A | CPD | 0.06 |  | 4.0E-02 | 1.3E-02 |  | 5.1E-02 | 1.6E-02 |  | 2.1E-02 | 2.5E-02 |  |
| rs7125588 | 11 | 113436072 | G | A | CPD | 0.43 |  | -6.2E-03 | 6.5E-03 |  | -1.1E-02 | 7.7E-03 |  | 7.8E-03 | 1.2E-02 | rs10750025, r^2^=0.14 |
| rs7951365 | 11 | 16377044 | C | T | CPD | 0.31 |  | -1.7E-03 | 6.7E-03 |  | -6.6E-03 | 8.0E-03 |  | -2.1E-03 | 1.2E-02 |  |
| rs10879871 | 12 | 75380511 | G | T | LSI | 0.65 |  | -9.9E-03 | 6.5E-03 |  | -8.7E-03 | 7.8E-03 |  | -1.9E-02 | 1.2E-02 |  |
| rs12831617 | 12 | 84758368 | T | C | LSI | 0.23 |  | 2.6E-02 | 7.4E-03 |  | 2.4E-02 | 8.8E-03 |  | 2.2E-02 | 1.3E-02 |  |
| rs7297175 | 12 | 56473808 | C | T | LSI | 0.59 |  | -3.8E-03 | 6.4E-03 |  | -4.1E-03 | 7.6E-03 |  | 3.7E-03 | 1.2E-02 |  |
| rs74086911 | 12 | 50015942 | A | G | LSI | 0.07 |  | -1.9E-02 | 1.2E-02 |  | -1.8E-02 | 1.4E-02 |  | -4.1E-02 | 2.2E-02 |  |
| rs6562474 | 13 | 67332812 | G | C | LSI | 0.36 |  | 1.2E-02 | 6.6E-03 |  | 9.5E-03 | 7.8E-03 |  | 2.6E-02 | 1.2E-02 |  |
| rs7333559 | 13 | 100546450 | A | G | LSI | 0.79 |  | -1.1E-02 | 8.3E-03 |  | -8.9E-03 | 9.9E-03 |  | -3.0E-02 | 1.5E-02 |  |
| rs3742365 | 14 | 104198251 | C | T | LSI | 0.41 |  | -1.1E-02 | 6.4E-03 |  | -7.5E-03 | 7.6E-03 |  | -1.5E-02 | 1.2E-02 |  |
| rs7155595 | 14 | 77502546 | C | A | LSI | 0.33 |  | 1.1E-02 | 6.7E-03 |  | 1.5E-02 | 7.9E-03 |  | 1.0E-02 | 1.2E-02 |  |
| rs860326 | 14 | 57342912 | T | C | LSI | 0.56 |  | 1.2E-03 | 6.7E-03 |  | 2.6E-03 | 8.0E-03 |  | -2.0E-02 | 1.2E-02 |  |
| rs11846838 | 14 | 104184737 | A | G | CPD | 0.33 |  | -5.1E-03 | 6.7E-03 |  | -5.1E-03 | 8.0E-03 |  | 5.8E-03 | 1.2E-02 |  |
| rs28485305 | 15 | 74044197 | T | C | LSI | 0.39 |  | -7.3E-03 | 6.4E-03 |  | -4.6E-03 | 7.6E-03 |  | -5.7E-03 | 1.2E-02 |  |
| rs35175834 | 15 | 47680815 | A | G | LSI | 0.22 |  | 6.6E-03 | 7.5E-03 |  | 1.2E-02 | 8.9E-03 |  | 7.0E-03 | 1.4E-02 |  |
| rs6598539 | 15 | 99204483 | C | T | LSI | 0.51 |  | 2.6E-03 | 6.5E-03 |  | -5.9E-03 | 7.7E-03 |  | -4.3E-03 | 1.2E-02 |  |
| rs8042134 | 15 | 97514404 | G | T | LSI | 0.45 |  | -6.2E-03 | 6.9E-03 |  | -6.4E-03 | 8.2E-03 |  | -8.5E-03 | 1.3E-02 |  |
| rs8042849 | 15 | 78817929 | T | C | LSI | 0.65 |  | -7.6E-03 | 6.5E-03 |  | -6.8E-03 | 7.7E-03 |  | -2.2E-03 | 1.2E-02 |  |
| rs10519203 | 15 | 78814046 | A | G | CPD | 0.66 |  | -7.0E-03 | 6.5E-03 |  | -5.9E-03 | 7.7E-03 |  | -1.4E-03 | 1.2E-02 |  |
| rs1115019 | 15 | 57141231 | C | T | CPD | 0.79 |  | -6.8E-03 | 7.6E-03 |  | -3.1E-03 | 9.1E-03 |  | -2.3E-02 | 1.4E-02 |  |
| rs182317 | 15 | 89943601 | T | G | CPD | 0.36 |  | 2.7E-03 | 6.6E-03 |  | -1.0E-04 | 7.9E-03 |  | -9.2E-03 | 1.2E-02 |  |
| rs3743063 | 15 | 79065171 | C | A | CPD | 0.59 |  | -1.0E-02 | 6.4E-03 |  | -1.3E-02 | 7.7E-03 |  | 1.2E-03 | 1.2E-02 |  |
| rs632811 | 15 | 59155050 | G | A | CPD | 0.34 |  | 6.0E-04 | 6.8E-03 |  | -7.4E-03 | 8.1E-03 |  | 1.7E-02 | 1.2E-02 |  |
| rs1050847 | 16 | 87443734 | T | C | LSI | 0.55 |  | 6.5E-03 | 6.6E-03 |  | 2.0E-03 | 7.8E-03 |  | 2.0E-02 | 1.2E-02 |  |
| rs11861214 | 16 | 746611 | T | G | LSI | 0.23 |  | 2.6E-03 | 7.3E-03 |  | 5.0E-04 | 8.7E-03 |  | -4.2E-03 | 1.3E-02 |  |
| rs12708665 | 16 | 24728227 | G | A | LSI | 0.71 |  | 5.5E-03 | 6.9E-03 |  | 1.6E-03 | 8.1E-03 |  | 2.0E-03 | 1.2E-02 | rs17177078, r^2^=0.21 |
| rs369230 | 16 | 89645437 | T | G | LSI | 0.71 |  | 1.8E-02 | 7.0E-03 |  | 1.8E-02 | 8.4E-03 |  | 1.5E-02 | 1.3E-02 |  |
| rs57611503 | 16 | 31165795 | A | G | LSI | 0.52 |  | 1.2E-02 | 6.6E-03 |  | 2.7E-03 | 7.8E-03 |  | 2.1E-02 | 1.2E-02 |  |
| rs60952428 | 16 | 75640521 | C | T | LSI | 0.09 |  | -4.0E-04 | 1.1E-02 |  | 1.2E-02 | 1.3E-02 |  | -9.0E-04 | 2.0E-02 |  |
| rs889398 | 16 | 69556715 | T | C | LSI | 0.43 |  | -1.4E-02 | 6.3E-03 |  | -1.3E-02 | 7.5E-03 |  | -3.1E-02 | 1.1E-02 |  |
| rs12924872 | 16 | 69552215 | T | C | CPD | 0.46 |  | -1.2E-02 | 6.4E-03 |  | -7.9E-03 | 7.6E-03 |  | -3.3E-02 | 1.2E-02 |  |
| rs1592485 | 16 | 52093549 | A | C | CPD | 0.61 |  | -6.0E-04 | 6.4E-03 |  | -3.6E-03 | 7.6E-03 |  | -7.0E-04 | 1.2E-02 |  |
| rs258321 | 16 | 89756473 | G | A | CPD | 0.44 |  | -2.5E-03 | 6.4E-03 |  | -4.0E-04 | 7.6E-03 |  | 1.7E-03 | 1.2E-02 |  |
| rs67596067 | 17 | 50333733 | A | G | LSI | 0.34 |  | -3.3E-03 | 6.7E-03 |  | 1.5E-03 | 8.1E-03 |  | 8.1E-03 | 1.2E-02 |  |
| rs732083 | 17 | 37834367 | A | G | LSI | 0.67 |  | 4.1E-03 | 6.6E-03 |  | 9.9E-03 | 7.8E-03 |  | -1.7E-02 | 1.2E-02 |  |
| rs8614 | 17 | 27588806 | A | C | LSI | 0.19 |  | 1.6E-02 | 8.6E-03 |  | 1.8E-02 | 1.0E-02 |  | 4.3E-03 | 1.6E-02 |  |
| rs9904288 | 17 | 47031973 | C | T | LSI | 0.29 |  | -3.4E-03 | 6.9E-03 |  | -2.3E-03 | 8.2E-03 |  | -1.3E-02 | 1.3E-02 |  |
| rs12967855 | 18 | 35138245 | G | A | LSI | 0.67 |  | -5.9E-03 | 6.9E-03 |  | -6.2E-03 | 8.3E-03 |  | 3.3E-03 | 1.3E-02 |  |
| rs62098013 | 18 | 50863861 | A | G | LSI | 0.38 |  | 4.6E-03 | 6.5E-03 |  | 3.6E-03 | 7.7E-03 |  | 4.5E-03 | 1.2E-02 |  |
| rs71367545 | 18 | 77576337 | A | G | LSI | 0.19 |  | -6.8E-03 | 8.7E-03 |  | -1.6E-02 | 1.1E-02 |  | 1.0E-03 | 1.6E-02 |  |
| rs4144686 | 18 | 53251725 | A | G | CPD | 0.17 |  | -7.1E-03 | 8.2E-03 |  | -1.7E-02 | 9.8E-03 |  | 5.8E-03 | 1.5E-02 |  |
| rs4485470 | 18 | 62125063 | A | G | CPD | 0.59 |  | -2.0E-03 | 6.3E-03 |  | -4.2E-03 | 7.6E-03 |  | -1.0E-04 | 1.2E-02 |  |
| rs35343344 | 19 | 18471610 | A | C | LSI | 0.25 |  | 1.5E-03 | 7.4E-03 |  | 6.4E-03 | 8.8E-03 |  | 2.2E-03 | 1.3E-02 |  |
| rs76608582 | 19 | 4474725 | A | C | LSI | 0.05 |  | -2.7E-02 | 1.9E-02 |  | -1.6E-02 | 2.3E-02 |  | -5.1E-02 | 3.5E-02 |  |
| rs117824460 | 19 | 41371480 | G | A | CPD | 0.02 |  | 1.3E-02 | 2.5E-02 |  | 2.0E-03 | 3.0E-02 |  | 4.9E-02 | 4.5E-02 |  |
| rs143200968 | 19 | 41338847 | C | G | CPD | 0.02 |  | 2.8E-03 | 2.5E-02 |  | 1.0E-04 | 2.9E-02 |  | 8.5E-02 | 4.4E-02 |  |
| rs56113850 | 19 | 41353107 | C | T | CPD | 0.56 |  | -1.0E-02 | 7.2E-03 |  | -1.6E-02 | 8.7E-03 |  | -2.2E-03 | 1.3E-02 |  |
| rs59208569 | 19 | 4044424 | C | G | CPD | 0.82 |  | 1.6E-02 | 8.8E-03 |  | 1.7E-02 | 1.1E-02 |  | -1.6E-03 | 1.6E-02 |  |
| rs8192726 | 19 | 41354496 | A | C | CPD | 0.07 |  | -1.2E-02 | 1.4E-02 |  | -8.4E-03 | 1.7E-02 |  | -2.7E-02 | 2.6E-02 |  |
| rs12481282 | 20 | 44761377 | C | G | LSI | 0.27 |  | -4.1E-03 | 7.1E-03 |  | -1.1E-02 | 8.4E-03 |  | 1.3E-02 | 1.3E-02 |  |
| rs348809 | 20 | 59032097 | G | A | LSI | 0.65 |  | 2.5E-02 | 7.0E-03 |  | 2.5E-02 | 8.4E-03 |  | 3.7E-02 | 1.3E-02 |  |
| rs4814873 | 20 | 19616429 | T | C | LSI | 0.24 |  | -6.9E-03 | 7.3E-03 |  | -9.5E-03 | 8.7E-03 |  | -1.5E-02 | 1.3E-02 |  |
| rs6011779 | 20 | 61984317 | T | C | LSI | 0.80 |  | 1.8E-02 | 8.2E-03 |  | 1.4E-02 | 9.7E-03 |  | 3.5E-02 | 1.5E-02 |  |
| rs6119897 | 20 | 31145415 | A | G | LSI | 0.25 |  | 2.0E-02 | 7.3E-03 |  | 2.6E-02 | 8.7E-03 |  | 2.7E-02 | 1.3E-02 |  |
| rs1737894 | 20 | 31054702 | G | C | CPD | 0.41 |  | 2.2E-02 | 6.3E-03 |  | 2.9E-02 | 7.6E-03 |  | 1.8E-02 | 1.2E-02 |  |
| rs2273500 | 20 | 61986949 | C | T | CPD | 0.15 |  | -1.4E-02 | 9.4E-03 |  | -8.7E-03 | 1.1E-02 |  | -2.3E-02 | 1.7E-02 |  |
| rs6078373 | 20 | 11863500 | A | G | CPD | 0.40 |  | -5.7E-03 | 6.8E-03 |  | 2.0E-03 | 8.1E-03 |  | -2.4E-02 | 1.2E-02 |  |
| rs147412694 | 21 | 40702786 | A | G | LSI | 0.14 |  | 1.6E-02 | 9.1E-03 |  | 2.1E-02 | 1.1E-02 |  | 1.6E-03 | 1.7E-02 |  |
| rs2838834 | 21 | 46665208 | T | C | LSI | 0.29 |  | 2.7E-03 | 6.8E-03 |  | -1.7E-03 | 8.1E-03 |  | 1.1E-03 | 1.2E-02 |  |
| rs7281463 | 21 | 40520783 | C | A | CPD | 0.41 |  | 1.5E-02 | 6.3E-03 |  | 1.6E-02 | 7.5E-03 |  | 1.3E-02 | 1.2E-02 |  |
| rs136233 | 22 | 31212410 | G | A | LSI | 0.19 |  | -5.0E-04 | 8.5E-03 |  | -1.0E-04 | 1.0E-02 |  | -9.0E-04 | 1.5E-02 |  |
| rs202645 | 22 | 41798520 | G | A | LSI | 0.80 |  | 5.3E-03 | 7.7E-03 |  | -7.3E-03 | 9.2E-03 |  | 3.1E-02 | 1.4E-02 | rs9607814, r^2^=0.97 |

^a^ Effect allele frequency in BCAC control; ^b^ Linkage disequilibrium based on 1000 Genome project European population.

Abbreviation: BC, breast cancer; ER, oestrogen receptor; EA, effect alleles; OA, other alleles; EAF, effect allele frequency; Chr, chromosome; SE, standard error; CPD, cigarettes per day; LSI; lifetime smoking; LD, linkage disequilibrium; AC, alcohol consumption.

## **Table S3. Statistical power of the study for detecting different odds ratios at 5% type I error.**

| **Cancer type** | **Variance explained (%)** | **Sample size** | **proportion of cases** | **OR ^a,b^ 1.20** | **OR ^a,b^ 1.15** | **OR ^a,b^ 1.10** | **OR ^a,b^ 1.05** |
| --- | --- | --- | --- | --- | --- | --- | --- |
| **CPD** |  |  |  |  |  |  |  |
| **Invasive BC** | 1.09% | 52349 | 0.5 | 0.59 | 0.39 | 0.21 | 0.09 |
| **ER positive** | 1.09% | 44063 | 0.4 | 0.51 | 0.33 | 0.18 | 0.08 |
| **ER negative** | 1.09% | 30066 | 0.13 | 0.22 | 0.15 | 0.09 | 0.06 |
| **LSI** |  |  |  |  |  |  |  |
| **Invasive BC** | 0.36% | 200753 | 0.54 | 0.68 | 0.46 | 0.25 | 0.10 |
| **ER positive** | 0.36% | 162470 | 0.43 | 0.60 | 0.39 | 0.21 | 0.09 |
| **ER negative** | 0.36% | 109400 | 0.16 | 0.29 | 0.19 | 0.11 | 0.07 |

^a^ Assumed odds ratio per standard deviation of the exposure variable and 5 % alpha level;^1,2^ ^b^ Fixed-effect meta-analysed between iCOGS and OncoArray

Calculations were performed using the online power calculator for Mendelian Randomization based on the publication by Burgess et al. (2014)^3^

Abbreviation: OR, odd ratio; SE, standard error; BC, breast cancer; ER, oestrogen receptor; CPD, cigarettes per day; LSI; lifetime smoking index

## **Table S4. The associations between instruments and well-known risk factors of breast cancer.**

| **Traits** |  | **wPRS-CPD** | | | |  | **wPRS-LSI** | | | |
| --- | --- | --- | --- | --- | --- | --- | --- | --- | --- | --- |
|  |  | **iCOGS** | | **OncoArray** | |  | **iCOGS** | | **OncoArray** | |
|  |  | **Summary Effect** | ***p*** | **Summary Effect** | ***p*** |  | **Summary Effect** | ***p*** | **Summary Effect** | ***p*** |
| Parous (yes) |  | -6.0E-03 | 1.3E-02 | -2.2E-03 | 1.9E-01 |  | 9.8E-04 | 3.9E-01 | 1.9E-03 | 4.6E-02 |
| Ever breastfeeding (yes) |  | -7.6E-03 | 1.7E-04 | -1.1E-03 | 4.6E-01 |  | -2.3E-03 | 3.2E-02 | -3.7E-05 | 9.7E-01 |
| Family history of breast cancer (yes) |  | -2.4E-04 | 9.2E-01 | -1.9E-03 | 2.8E-01 |  | 6.0E-04 | 5.9E-01 | 4.3E-04 | 6.5E-01 |
| Postmenopausal status (yes) |  | -8.8E-04 | 6.4E-01 | 5.2E-04 | 7.3E-01 |  | 1.4E-04 | 8.8E-01 | 2.1E-03 | 1.0E-02 |
| Ever use of MHT |  | -7.1E-04 | 6.8E-01 | 5.8E-04 | 6.6E-01 |  | -7.4E-04 | 4.9E-01 | 5.3E-04 | 4.9E-01 |
| Age at menarche (years) |  | -1.8E-04 | 7.4E-01 | -1.6E-04 | 6.9E-01 |  | -8.1E-05 | 7.8E-01 | -2.5E-04 | 2.6E-01 |
| Age at menopause (years) |  | -1.6E-04 | 4.1E-01 | -1.3E-04 | 3.1E-01 |  | -1.9E-04 | 8.6E-02 | -3.1E-04 | 3.9E-05 |
| Age at first live birth (years) |  | -7.0E-04 | 1.3E-04 | -1.8E-04 | 1.9E-01 |  | -1.3E-04 | 3.3E-03 | -8.3E-04 | 3.1E-25 |
| Parity (numbers) |  | -7.3E-04 | 2.9E-01 | 9.5E-05 | 8.4E-01 |  | 7.3E-04 | 2.4E-02 | 7.8E-04 | 2.1E-03 |
| BMI (kg/m^2^) among premenopausal women |  | -9.7E-06 | 9.8E-01 | -2.0E-04 | 4.0E-01 |  | 7.9E-04 | 2.6E-06 | 3.4E-04 | 6.6E-03 |
| BMI (kg/m^2^) among postmenopausal women |  | -1.4E-04 | 5.0E-01 | 5.7E-05 | 7.0E-01 |  | 2.2E-04 | 5.5E-02 | 4.1E-04 | 7.0E-07 |
| Education level ^a^  Secondary education  Tertiary education |  | -2.6E-03  -4.8E-03 | 3.8E-01  1.3E-01 | -1.3E-03  -2.6E-03 | 5.6E-01  2.9E-01 |  | -1.4E-04  -3.4E-03 | 9.4E-01  1.0E-01 | -2.4E-03  -1.0E-02 | 8.4E-02  3.5E-12 |
| Frequency alcoholic drinks (glasses / week) |  | 3.3E-05 | 5.2E-01 | -5.3E-05 | 4.7E-01 |  | 2.4E-05 | 5.4E-01 | 1.9E-05 | 7.1E-01 |

^a^ Primary education used as reference

Abbreviation: wPRS, weighted polygenic risk score; CPD, cigarettes per day; LSI, lifetime smoking index; MHT, menopausal hormone therapy.

## **Table S5. Association of cigarette smoke with breast cancer risk according to ER status: results from the two-sample Mendelian randomization estimate using summary statistics**

|  | **ER positive** | | | | | | |  | **ER negative** | | | | | | |
| --- | --- | --- | --- | --- | --- | --- | --- | --- | --- | --- | --- | --- | --- | --- | --- |
|  | **CPD** | | |  | **LSI** | | |  | **CPD** | | |  | **LSI** | | |
|  | **OR ^i^** | **95% CI** | ***p*** |  | **OR ^i^** | **95% CI** | ***p*** |  | **OR ^i^** | **95% CI** | ***p*** |  | **OR ^i^** | **95% CI** | ***p*** |
| **IVW ^a^** | 1.03 | 0.88 - 1.21 | 0.71 |  | 1.14 | 1.00 - 1.30 | 4.5×10^-02^ |  | 1.04 | 0.88 - 1.22 | 0.64 |  | 1.14 | 0.95 - 1.37 | 0.17 |
| **Multivariable IVW ^a,b^** | 1.05 | 0.90 - 1.23 | 0.55 |  | 1.50 | 1.14 - 1.97 | 4.1×10^-03^ |  | 0.98 | 0.84 - 1.15 | 0.84 |  | 1.30 | 0.87 - 1.94 | 0.20 |
| **IVW after outlier corrected ^a,c^** | 1.03 | 0.92 - 1.17 | 0.59 |  | - | - | - |  | - | - | - |  | 1.19 | 1.00 - 1.42 | 4.8×10^-02^ |
| **Egger regression ^a,d^** | 1.00 | 0.76 - 1.32 | 0.98 |  | 1.70 | 1.02 - 2.83 | 4.0×10^-02^ |  | 1.07 | 0.81 - 1.42 | 0.63 |  | 0.65 | 0.32 - 1.35 | 0.25 |
| **Multivariable Egger regression ^a,b,e^** | 0.99 | 0.76 - 1.30 | 0.96 |  | 1.67 | 1.02 - 2.72 | 4.2×10^-02^ |  | 0.95 | 0.73 - 1.25 | 0.74 |  | 0.64 | 0.31 - 1.28 | 0.21 |
| **Weighted Median estimator ^a,f^** | 1.06 | 0.91 - 1.24 | 0.42 |  | 1.13 | 0.98 - 1.30 | 8.4×10^-02^ |  | 0.99 | 0.79 - 1.24 | 0.92 |  | 1.21 | 0.97 - 1.50 | 9.1×10^-02^ |
| **Two MR-RAPS estimator ^a,g^** | 1.03 | 0.93 - 1.14 | 0.53 |  | 1.42 | 1.26 - 1.60 | 1.2×10^-08^ |  | 1.04 | 0.88 - 1.23 | 0.61 |  | 1.39 | 0.45 - 4.30 | 0.57 |
| **Weighted mode estimator ^a,h^** | 1.05 | 0.90 - 1.21 | 0.56 |  | 1.28 | 0.88 - 1.86 | 0.20 |  | 1.00 | 0.85 - 1.19 | 0.95 |  | 1.17 | 0.67 - 2.07 | 0.58 |

All two-sample MR analyses using summary-level data were performed in oestrogen receptor (ER)-positive (70,435 cases/ 88,386 controls) and ER-negative tumours (17,365 cases/ 88,386 controls) regardless of smoking status; ^a^ Estimates derived using summary statistics;^4^ ^b^ Multivariable analysis after adjusting for genetically predicted alcohol consumption (drinks per week), body mass index, and education attainment by using summary-level data from GWAS outcome (alcohol assumption,^2^ body mass index (BMI) among female,^5^ and education attainment;^6^ ^c^ The MR pleiotropy residual sum and outlier test (MR-PRESSO) was implemented to identify outlying genetic variants (rs11940255, rs1737894, and rs73229090 for CPD on ER-positive tumor, rs2867112 for LSI on ER-negative tumor) and analyses were re-run after excluding these variants.^7^ No outlying genetic variant was detected for CPD on ER-negative tumor; ^d^ The MR-Egger intercept yielded no indication of strong pleiotropic effects (CPD on ER positive tumor: β_0_ = -1.1E-03, *p* = 0.77; LSI on ER positive tumour: β_0_ = -6.1E-03, *p* = 0.11; CPD on ER negative tumor: β_0_ = -1.0E-03, *p* = 0.79; LSI on ER negative tumour: β_0_= 8.6E-03, *p*= 0.12); ^e^ The multivariable MR-Egger intercept yielded no indication of strong pleiotropic effects (CPD on ER positive tumor: β_0_ = 1.93E-03, *p* = 0.61; LSI on ER positive tumour: β_0_ = -2.3E-03, *p* = 0.60; CPD on ER negative tumor: β_0_ = 9.7E-0, *p* = 0.80; LSI on ER negative tumour: β_0_ = 1.5E-02, *p* = 0.02); ^f^ Estimates derived using weighted median estimator approach, where 50% of the variants included in each genetic instrument are assumed to be invalid;^8^ ^g^ Estimates derived using Mendelian randomization robust adjusted proﬁle score (MR-RAPS) method;^9 h^ Estimates derived using weighted mode estimator approach, where the largest group of instruments with consistent MR estimate are assumed to be valid;^10^ ^i^ OR per standard deviation (SD).

Abbreviation: OR, odd ratio; MR, Mendelian randomization; ER, oestrogen receptor; IVW, inverse-variance weighted; CI: Confidential interval; CPD, cigarettes per day; LSI, lifetime smoking index; RAPS, robust adjusted proﬁle score; *p*, p-value.

## **Table S6. Associations of genetic risk scores for additional cigarette smoke exposure-related traits with breast cancer risk: results from Mendelian randomization analysis**

|  |  | **Age at SI ^c,e^** | | |  | **SI ^d,f^** | | |  | **SC ^c,g^** | | |
| --- | --- | --- | --- | --- | --- | --- | --- | --- | --- | --- | --- | --- |
|  |  | **OR** | **95% CI** | ***p*** |  | **OR** | **95% CI** | ***p*** |  | **OR** | **95% CI** | ***p*** |
| **Model 1 ^a^** |  | 0.88 | 0.25 - 3.07 | 0.84 |  | 1.05 | 0.99 - 1.11 | 0.08 |  | 1.06 | 0.88 - 1.27 | 0.52 |
| **Model 2 ^b^** |  | 0.63 | 0.14 - 2.86 | 0.55 |  | 1.04 | 0.97 - 1.12 | 0.24 |  | 1.16 | 0.92 - 1.48 | 0.21 |

^a^ Adjusted for age, sex and top 10 PCs. For analyses of SI, all participants (108,420 cases and 87,681 controls) were included, whereas ones for age at SI and SC, the analyses were restricted to only smokers (26,147 cases and 26,072 controls). *P*-value for heterogeneity between iCOGS and OncoArray data of age at SI, SI, and SC are 0.70, 0.40, and 0.21, respectively; ^b^ Additionally adjusted for ever breastfeeding, postmenopausal status, age at menopause, BMI, age at first live birth, parity, and education level. For analyses of SI, all participants (17,936 cases and 16,654 controls) were included, whereas ones for age at SI and SC, the analyses were restricted to only smokers (7,360 cases and 7,168 controls). *P-* value for heterogeneity between iCOGS and OncoArray data of age at SI, SI, and SC are 0.27, 0.43, and 0.12, respectively; ^c^ Analyses restricted to only smoker; ^d^ Analyses were conducted using all participants (including ever, never smokers); ^e^ OR per year; ^f^ Dichotomous trait; ever-smokers vs. non-smokers; ^g^ Dichotomous trait; current smokers vs. formal smokers.

Abbreviations: No., number; SI, smoking initiation; SC, smoking cessation; CI, confidence interval; OR, odds ratio per year; BMI, body mass index; *p*, p-value

## **Table S7. Associations of genetic risk scores including alcohol consumption associated SNPs for cigarette smoke exposure-related traits with breast cancer risk: results from Mendelian randomization analysis**

|  |  | **CPD** | | | |  | **LSI** | | | |
| --- | --- | --- | --- | --- | --- | --- | --- | --- | --- | --- |
|  |  | **No. of cases / controls** | **OR ^d^** | **95% CI** | ***p*** |  | **No. of cases / controls** | **OR ^e^** | **95% CI** | ***p*** |
| **Model 1** **^a^** |  | 26,147 / 26,072 | 1.03 | 0.90 - 1.17 | 0.69 |  | 108,420 / 87,681 | 1.15 | 1.04 - 1.26 | 4.6×10^-3^ |
| **Model 2 ^b^** |  | 7,360 / 7,168 | 0.98 | 0.82 - 1.19 | 0.89 |  | 17,936 / 16,654 | 1.24 | 1.07 - 1.44 | 5.2×10^-3^ |
| **Model 3 ^c^** |  | 2,892 / 2,754 | 0.82 | 0.57 - 1.17 | 0.28 |  | 7,716 / 7,028 | 1.12 | 0.86 - 1.47 | 0.41 |

^a^ Adjusted for age, sex and top 10 PCs. *P*-value for heterogeneity between iCOGS and OncoArray data of cigarettes per day and lifetime smoking index are 0.37, 0.16 respectively; ^b^ In addition to adjustment of Model 1, additionally adjusted for ever breastfeeding, postmenopausal status, age at menopause, BMI, age at first live birth, parity, and education level. *P-* value for heterogeneity between iCOGS and OncoArray data of cigarettes per day and lifetime smoking index are 0.38, 0.42 respectively; ^c^ In addition to adjustment of Model 2, additionally adjusted for alcohol assumption (glasses per day). *P-* value for heterogeneity between iCOGS and OncoArray data of cigarettes per day and lifetime smoking index are 0.21, 0.80 respectively; ^d^ OR per pack of cigarettes per day; ^e^ OR per standard deviation.

Abbreviations: No, number; CPD, cigarettes per day; LSI, lifetime smoking index; CI, confidence interval; OR, odds ratio per year; BMI, body mass index; *p*, p-value.

## **Table S8. Ethical review boards providing approval for each BCAC study.**

| **Study** | **Acronym** | **Country** | **Approval Committee(s)** |
| --- | --- | --- | --- |
| The Two Sister Study | 2SISTER | USA | Institutional Review Board of the National Institute of Environmental Health Sciences; NIH and the Copernicus Group Independent Review Board |
| Australian Breast Cancer Family Study | ABCFS | Australia | The University of Melbourne Health Sciences Human Ethics Sub-Committee (HESC) |
| Amsterdam Breast Cancer Study | ABCS | Netherlands | Leiden University Medical Center (LUMC) Commissie Medische Ethiek; Protocol Toetsingscommissie van Het Nederlands Kanker Instituut-Antoni van Leeuwenhoek Ziekenhuis |
| Amsterdam Breast Cancer Study - Familial | ABCS-F | Netherlands | Leiden University Medical Center (LUMC) Commissie Medische Ethiek; Protocol Toetsingscommissie van Het Nederlands Kanker Instituut-Antoni van Leeuwenhoek Ziekenhuis |
| Australian Breast Cancer Tissue Bank | ABCTB | Australia | Sydney Local Health District (RPA Zone) Research Ethics and Governance Office |
| Agricultural Health Study | AHS | USA | National Institute of Health (NIH) Population Sciences IRB |
| Bavarian Breast Cancer Cases and Controls | BBCC | Germany | Friedrich-Alexander-Universitat Erlangen-Nurnberg Medizinische Fakultat Ethik-Commission |
| British Breast Cancer Study | BBCS | UK | South East Multi-Centre Research Ethics Committee |
| Breast Cancer Environment and Employment Study | BCEES | Australia | The University of Western Australia Human Ethics Research Committee |
| New York Breast Cancer Family Registry | BCFR-NY | USA | Columbia University Institutional Review Board |
| Philadelphia Breast Cancer Family Registry | BCFR-PA | USA | Institutional Review Board Fox Chase Cancer Center |
| Utah Breast Cancer Family Registry | BCFR-UTAH | USA | University of Utah Institutional Review Board |
| Breast Cancer In Northern Israel Study | BCINIS | Israel | Carmel Medical Center |
| Breast Cancer In Galway Genetic Study | BIGGS | Ireland | Galway University College Hospital Clinical Research Ethical Committee |
| Breast Oncology Galicia Network | BREOGAN | Spain | Comité Autonómico de Ética de la Investigación de Galicia |
| Breast Cancer Study of the University Clinic Heidelberg | BSUCH | Germany | Ethikkommission Medizinische Fakultat Heidelberg, University of Heidelberg |
| Canadian Breast Cancer Study | CBCS | Canada | University of British Columbia - BC Cancer Research Ethics Board; Queen’s University Health Sciences and Affiliated Teaching Hospitals Research Human Ethics Board (HSREB) |
| Crete Cancer Genetics Program | CCGP | Greece | Epistimoniko Symvoulio (Scientific Council of the University General hospital of Heraklion) |
| CECILE Breast Cancer Study | CECILE | France | Comité Consultatif de Protection des Personnes dans la Recherche Biomédicale de Bicêtre (Le Kremlin-Bicêtre FR-94270) |
| Copenhagen General Population Study | CGPS | Denmark | Kobenhavns Amt den Videnskabsetiske Komite (Scientific ethical committee, Copenhagen County) |
| Spanish National Cancer Centre Breast Cancer Study | CNIO-BCS | Spain | Comité de ética de la Investigacion y de Bienestrar Animal del Insituto de Salud Carlos III |
| Cancer Prevention Study-II Nutrition Cohort | CPSII | USA | Emory University Institutional Review Board |
| California Teachers Study | CTS | USA | UC Irvine: Office of Research Institutional Review Board |
| DietCompLyf Breast Cancer Survival Study | DIETCOMPLYF | UK | MREC Committee A and NRES Committee London –Bentham |
| European Prospective Investigation Into Cancer and Nutrition | EPIC | Various within EU | Institutional Review Board of the International Agency for Research on Cancer, Lyon, France; Ethics Commission of the Faculty of Medicine of the University of Heidelberg |
| ESTHER Breast Cancer Study | ESTHER | Germany | Ruprecht-Karls-Universitat Medizinische Fakultat Heidelberg Ethikkommission |
| Family History Risk Study | FHRISK | UK | NRES Committee North West - Greater Manchester Central |
| German Consortium for Hereditary Breast & Ovarian Cancer | GC-HBOC | Germany | Ethik-Kommission der Medizinischen Fakultat der Universitat zu Koln |
| Gene Environment Interaction and Breast Cancer in Germany | GENICA | Germany | Ethikkommission Rheinische Friedrich-Wilhels-Universität Bonn |
| A randomized phase II trial investigating the addition of carboplatin to neoadjuvant therapy for triple-negative and HER2-positive early breast cancer | GEPARSIXTO | Germany | Ethikkommission der Ärztkammer Nordrhein |
| Genetic Epidemiology Study of Breast Cancer by Age 50 | GESBC | Germany | Medizinische Fakultat Heidelberg Ethikkommission |
| Hannover Breast Cancer Study | HABCS | Germany | Medizinische Hochschule Hannover Ethik-Kommission |
| Hospital Clinico San Carlos | HCSC | Spain | Hospital Clínico San Carlos Ethical Committee |
| Helsinki Breast Cancer Study | HEBCS | Finland | Helsingin ja uudenmaan sairaanhoitopiiri (Helsinki University Hospital Ethics Committee) |
| Hannover-Minsk Breast Cancer Study | HMBCS | Belarus | Medizinische Hochschule Hannover Ethik-Kommission |
| Hannover-Ufa Breast Cancer Study | HUBCS | Russia | Ethical Committee of Institute of Biochemistry and Genetics, Ufa Scientific Center of Russian Academy of Sciences |
| Karolinska Breast Cancer Study | KARBAC | Sweden | Regionala Etikprovningsnamnden i Stockholm (Regional Ethical Review Board in Stockholm) |
| Karolinska Mammography Project for Risk Prediction of Breast Cancer - Cohort Study | KARMA | Sweden | Regionala Etikprovningsnamnden i Stockholm (Regional Ethical Review Board in Stockholm) |
| Kuopio Breast Cancer Project | KBCP | Finland | Pohjois-Savon Sairraanhoitopiirin Kuntayhtyma Tutkimuseettinen Toimikunta |
| Kathleen Cuningham Foundation Consortium for Familial Breast Cancer/Australian Ovarian Cancer Study | kConFab/AOCS | Australia | kConFab: Peter MacCallum Cancer Centre Ethics Committee |
|  |  |  | AOCS: Peter MacCallum Cancer Centre Ethics Committee |
| Leuven Multidisciplinary Breast Centre | LMBC | Belgium | Commissie Medische Ethiek van de Universitaire Ziekenhuizen Kuleuven |
| Macedonian Breast Cancer Study | MABCS | Republic of North Macedonia | Ethic Subcommittee of Medicine, Pharmacy, Veterinary Medicine and Dentistry, Macedonia Academy of Sciences and Arts |
| Mammary Carcinoma Risk Factor Investigation | MARIE | Germany | Medizinische Fakultat Heidelberg Ethikkommission; Ethik-Kommission der Arztekammer Hamburg |
| Milan Breast Cancer Study Group | MBCSG | Italy | Comitato Etico Indipendente della Fondazione IRCCS "Istituto Nazionale dei Tumori" |
| Mayo Clinic Breast Cancer Study | MCBCS | USA | Mayo Clinic IRB |
| Melbourne Collaborative Cohort Study | MCCS | Australia | The Cancer Council Victoria Human Research Ethics Committee |
| Multi-ethnic Cohort | MEC | USA | University of Southern California Health Sciences Campus IRB |
| Melanoma Inquiry of Southern Sweden | MISS | Sweden | Regional Ethical Board in South Sweden |
| Mayo Mammography Health Study | MMHS | USA | Mayo Clinic IRB |
| Memorial Sloan-Kettering Cancer Center | MSKCC | USA | Memorial Sloan-Kettering Cancer Center IRB/Privacy Board-B |
| Montreal Gene-Environment Breast Cancer Study | MTLGEBCS | Canada | McGill University IRB |
| Norwegian Breast Cancer Study | NBCS | Norway | Regionale Komitere for Medisinsk og Helsefaglig Forskningsetikk |
| Nashville Breast Health Study | NBHS | USA | Vanderbilt University Medical Center IRB |
| Northern California Breast Cancer Family Registry | NC-BCFR | USA | Stanford University IRB |
| North Carolina Breast Cancer Study | NCBCS | USA | Office of Human Research Ethics, the University of North Carolina, Chapel Hill |
| Nurses Health Study | NHS | USA | Partners Human Research, Partners Healthcare System (PHS) IRB |
| Nurses Health Study 2 | NHS2 | USA | Partners Human Research, Partners Healthcare System (PHS) IRB |
| Oulu Breast Cancer Study | OBCS | Finland | Pohjois-Pohjenmaan Sairaanhoitopiirin Kuntayhtymä: Alueellinen Eettinen Toimikunta; Terveyden Ja Hyvinvoinnin Laitos (Finnish National Institute For Health And Welfare) |
| Ontario Familial Breast Cancer Registry | OFBCR | Canada | Mount Sinai Hospital Research Ethics Board |
| Leiden University Medical Centre Breast Cancer Study | ORIGO | Netherlands | Medical Ethical Committee and Board of Directors of the Leiden University Medical Center (LUMC) |
| NCI Polish Breast Cancer Study | PBCS | Poland | National Cancer Institute Special Studies Institutional Review Board (NCI-SSIRB) |
| Karolinska Mammography Project for Risk Prediction of Breast Cancer - Case-Control Study | pKARMA | Sweden | Regionala Etikprovningsnamnden i Stockholm (Regional Ethical Review Board in Stockholm) |
| The Prostate, Lung, Colorectal and Ovarian (PLCO) Cancer Screening Trial | PLCO | USA | National Cancer Institute Special Studies Institutional Review Board (NCI-SSIRB) |
| Prospective Study of Outcomes in Sporadic Versus Hereditary Breast Cancer | POSH | UK | South West Multi-centre Research Ethics Committee |
| Evaluation of Predictive Factors regarding the Effectivity of Aromatase Inhibitor Therapy | PREFACE | Germany | Friedrich-Alexander-Universitat Erlangen-Nurnberg Medizinische Fakultat Ethik-Commission |
| Predicting the Risk Of Cancer At Screening Study | PROCAS | UK | NRES Committee North West - Greater Manchester Central |
| Rotterdam Breast Cancer Study | RBCS | Netherlands | Medische Ethische Toetsings Commissie Erasmus Medisch Centrum |
| Singapore and Sweden Breast Cancer Study | SASBAC | Sweden | Regionala Etikprovningsnamnden i Stockholm (Regional Ethical Review Board in Stockholm) |
| Sheffield Breast Cancer Study | SBCS | UK | Yorkshire & The Humber - Sheffield Research Ethics Committee |
| Study of Epidemiology and Risk factors in Cancer Heredity | SEARCH | UK | Multi Centre Research Ethics Committee (MREC) |
| The Sister Study | SISTER | USA | Institutional Review Board of the National Institute of Environmental Health Sciences; NIH and the Copernicus Group Independent Review Board |
| Städtisches Klinikum Karlsruhe Deutsches Krebsforschungszentrum Study | SKKDKFZS | Germany | Medizinische Fakultat Heidelberg Ethikkommission |
| Swedish Mammography Cohort | SMC | Sweden | Regionala Etikprovningsnamnden i Stockholm (Regional Ethical Review Board in Stockholm) |
| Simultaneous Study of Gemcitabine-Docetaxel Combination adjuvant treatment | SUCCESSB | Germany | Ethikkommission der Medizinischen Fakultät der Ludwig-Maximilians-Universität München |
| Simultaneous Study of Docetaxel Based Anthracycline Free Adjuvant Treatment Evaluation | SUCCESSC | Germany | Ethikkommission der Medizinischen Fakultät der Heinrich Heine Universität Düsseldorf |
| IHCC-Szczecin Breast Cancer Study | SZBCS | Poland | Komisji Bioetycznej Pomorskiej Akademii Medycznej |
| Triple Negative Breast Cancer Consortium Study | TNBCC | Various | Mayo Clinic IRB |
| UCI Breast Cancer Study | UCIBCS | USA | UC Irvine: Office of Research Institutional Review Board |
| UK Breakthrough Generations Study | UKBGS | UK | London- South East Research Ethics Committee |
| United Kingdom Ovarian cancer Population Study | UKOPS | UK | NRES Committee London – Harrow |
| US Radiologic Technologists Study | USRT | USA | National Cancer Institute Special Studies Institutional Review Board (NCI-SSIRB) |

## **Figure S1. Associations of genetic risk scores for cigarette smoke exposure-related traits with overall breast cancer risk: stratified analysis.**


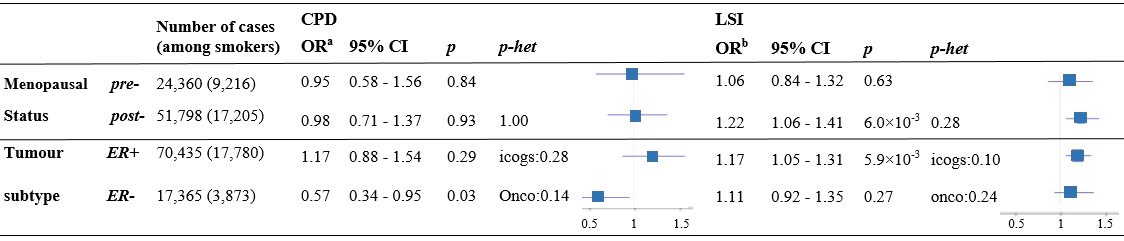
 All models were adjusted for age, study and top 10 PCs. The *p_het_* was obtained from heterogeneity test across strata. ^a^ OR of pack of cigarettes per day; ^b^ OR of standard deviation.

Abbreviations: CPD, cigarettes per day; LSI, lifetime smoking index; ER, oestrogen receptor; OR, odds ratio per year; CI, confidence interval; *p*, p-value; *p_het_,* p-value for heterogeneity.

**Figure S2. Scatter plot of IVW and sensitivity analyses of cigarettes per day (A) and lifetime smoking index (B) and overall breast cancer risk.**


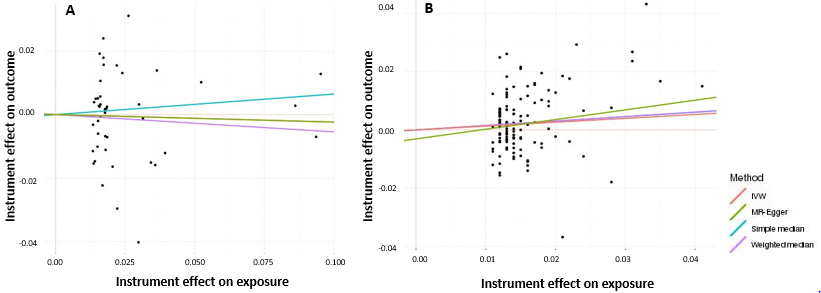


**Red line (inverse variance weighted, IVW):** Estimates derived using summary statistics.^4^ **Green line (MR-Egger**): Estimates derived from the slope of MR-Egger.^11^ The MR-Egger intercept yielded no indication of strong pleiotropic effects (CPD on overall breast cancer: β_0_ = 0.24E-04, *p* = 0.99; LSI on overall breast cancer: β_0_ = -3.0E-03, *p* = 0.39). **Blue and violet line (simple and weighted Median)**: Estimates derived using median estimator approach, where 50% of the variants included in each genetic instrument are assumed to be invalid.^8^ This plot was drawn using the R-package “MendelianRandomization”.^12^

## **Figure S3. Scatter plot of IVW and sensitivity analyses of cigarettes per day (A) and lifetime smoking index (B) and ER positive breast cancer risk.**


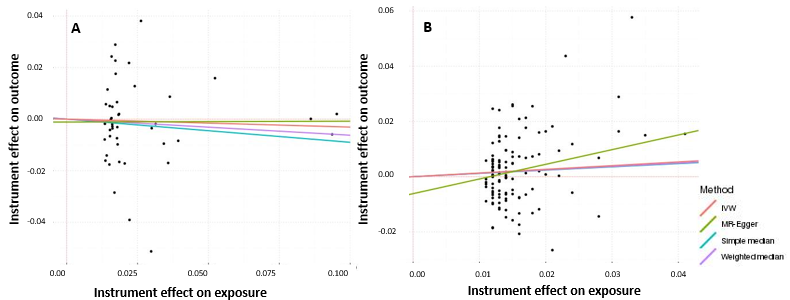


**Red line (inverse variance weighted, IVW):** Estimates derived using summary statistics.^4^ **Green line (MR-Egger**): Estimates derived from the slope of MR-Egger.^11^ The MR-Egger intercept yielded no indication of strong pleiotropic effects (CPD on ER positive tumor: β_0_ = -1.1E-03, *p* = 0.77; LSI on ER positive tumor: β_0_ = -6.0E-03, *p* = 0.18). **Blue and violet line (simple and weighted Median)**: Estimates derived using median estimator approach, where 50% of the variants included in each genetic instrument are assumed to be invalid.^8^ This plot was drawn using the R-package “MendelianRandomization”.^12^

## **Figure S4. Scatter plot of IVW and sensitivity analyses of cigarettes per day (A) and lifetime smoking index (B) and ER negative breast cancer risk.**


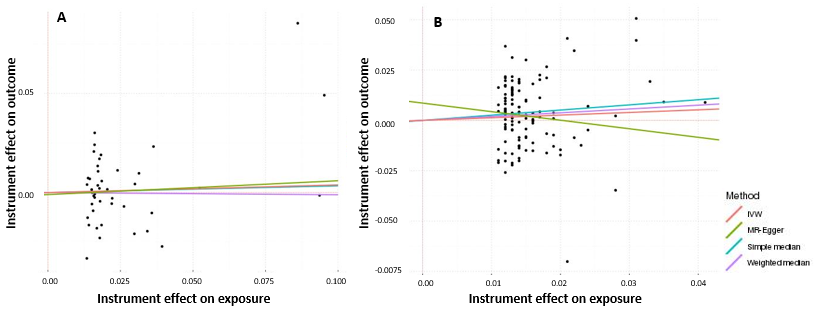


**Red line (inverse variance weighted, IVW):** Estimates derived using summary statistics.^4^ **Green line (MR-Egger**): Estimates derived from the slope of MR-Egger.^11^ The MR-Egger intercept yielded no indication of strong pleiotropic effects (CPD on ER negative tumour: β_0_ = -1.0E-03, *p* = 0.79; LSI on ER negative tumour: β_0_ = 7.98E-03, *p* = 0.16). **Blue and violet line (simple and weighted Median)**: Estimates derived using median estimator approach, where 50% of the variants included in each genetic instrument are assumed to be invalid.^8^ This plot was drawn using the R-package “MendelianRandomization”.^12^

## **Supplementary Note**

### ***Association analysis of wPGS for other smoking traits with invasive breast cancer risk.***

For further analyses, we included three smoking behaviours-related traits, age at smoking initiation (age at SI; quantitative trait; age at which an individual started smoking cigarettes regularly), smoking initiation (SI; dichotomous trait; ever-smokers vs. non-smokers) and smoking cessation (SC; dichotomous trait; current smokers vs. formal smokers). The recent GWAS of cigarettes per day from GSCAN consortium identified 55, 10, and 24 conditionally independent genome-wide significant SNPs, explaining 0.17%, and 0.10% of the variance in a sample of 341,427 and 547,219 ever-smokers of European ancestry for age at SI and SC, respectively.^2^ The GWAS also identified 375 SI-associated SNPs, explaining 2.32% of the variance in 1,232,091 individuals, including never and ever smokers.^2^ SNPs were selected if they were reported to be associated at the genome-wide significance level (p ≤ 5 × 10^-8^) and had a minor allele frequency (MAF) over 1% and in low linkage disequilibrium (LD) (r^2^ < 0.1). Lastly, we excluded SNPs correlated with any alcohol consumption associated-SNPs (r^2^ > 0.1) from recent large-scale GWAS (drinks per week, *p* ≤ 5 × 10^-8^).^2^ We included a total of 338, seven, and 22 variants associated with SI, age at SI, and SC, respectively. We generated weighted polygenic scores (wPGSs) using individual-level data of BCAC participants.

The model was adjusted for age (continuous), principal components (PCs) of genetic ancestry (first 10 PCs for iCOGS and OncoArray, separately), and study site. We additionally adjusted for ever breastfeeding, postmenopausal status, age at menopause, BMI, age at first live birth, parity, and education level in Model 2. Association analysis of the wPGSs with breast cancer risk using logistic regression was performed using fixed-effect meta-analyses combining iCOGS and OncoArray results. Analysis of SI was conducted using all participants (including ever, never smokers) of 108,420 cases (40,178 for iCOGS, 68,242 for OncoArray) and 87,681 controls (35,314 for iCOGS, 52,367 for OncoArray), whereas those of age SI and SC were performed solely in ever smokers, and based on 26,147 cases (7,342 for iCOGS, 18,805 for OncoArray) and 26,072 controls (8,489 for iCOGS, 17,583 for OncoArray).

Scaling was applied to convert the wPGSs for age at SI into meaningful units through dividing them by the linear regression coefficient of self-reported age at SI (0.58 per years old at smoking initiation). The regression coefficient of the smoking behaviours was derived from a meta-analysis of iCOGS and OncoArray data on smoking behaviours among 26,072 among ever smoker controls (8,490 for iCOGS and 17,703 for OncoArray).

### ***Association analysis of wPGS for alcohol use with invasive breast cancer risk.***

The recent GWAS from GSCAN consortium identified genome-wide significant SNPs related to alcohol use.^2^ For this analysis, SNPs were selected if they were reported to be associated at the genome-wide significance level (*p* ≤ 5 × 10^-8^) and had a MAF above or equal to 1%.^2^ After we filtered the list of alcohol use-associated SNPs so that the remaining SNPs were not in LD (r^2^ > 0.1), a total of 91 SNPs were included in our analysis. We generated wPGSs using individual-level data of BCAC participants (108,718 invasive breast cancer cases and 92,035 controls). Association analysis of the wPGSs with breast cancer risk using logistic regression was performed using fixed-effect meta-analyses combining iCOGS and OncoArray results. The model was adjusted for age (continuous), PCs of genetic ancestry (first 10 PCs for iCOGS and OncoArray, separately), and study site. We additionally adjusted for lifetime smoking index in 40,793 invasive breast cancer cases and 44,287 controls.

**Reference**

1. Wootton RE, Richmond RC, Stuijfzand BG, Lawn RB, Sallis HM, Taylor GMJ *et al.* Evidence for causal effects of lifetime smoking on risk for depression and schizophrenia: a Mendelian randomisation study. *Psychological medicine* 2019: 1-9; e-pub ahead of print 2019/11/07; doi 10.1017/s0033291719002678.

2. Liu M, Jiang Y, Wedow R, Li Y, Brazel DM, Chen F *et al.* Association studies of up to 1.2 million individuals yield new insights into the genetic etiology of tobacco and alcohol use. *Nat Genet* 2019; **51**(2): 237-244; e-pub ahead of print 2019/01/16; doi 10.1038/s41588-018-0307-5.

3. Burgess S. Sample size and power calculations in Mendelian randomization with a single instrumental variable and a binary outcome. *Int J Epidemiol* 2014; **43**(3): 922-929; e-pub ahead of print 2014/03/13; doi 10.1093/ije/dyu005.

4. Lawlor DA, Tilling K, Davey Smith G. Triangulation in aetiological epidemiology. *Int J Epidemiol* 2016; **45**(6): 1866-1886; e-pub ahead of print 2017/01/22; doi 10.1093/ije/dyw314.

5. Pulit SL, Stoneman C, Morris AP, Wood AR, Glastonbury CA, Tyrrell J *et al.* Meta-analysis of genome-wide association studies for body fat distribution in 694 649 individuals of European ancestry. *Human molecular genetics* 2019; **28**(1): 166-174; e-pub ahead of print 2018/09/22; doi 10.1093/hmg/ddy327.

6. Lee JJ, Wedow R, Okbay A, Kong E, Maghzian O, Zacher M *et al.* Gene discovery and polygenic prediction from a genome-wide association study of educational attainment in 1.1 million individuals. *Nature Genet* 2018; **50**(8): 1112-1121; doi 10.1038/s41588-018-0147-3.

7. Verbanck M, Chen CY, Neale B, Do R. Detection of widespread horizontal pleiotropy in causal relationships inferred from Mendelian randomization between complex traits and diseases. *Nat Genet* 2018; **50**(5): 693-698; e-pub ahead of print 2018/04/25; doi 10.1038/s41588-018-0099-7.

8. Bowden J, Davey Smith G, Haycock PC, Burgess S. Consistent Estimation in Mendelian Randomization with Some Invalid Instruments Using a Weighted Median Estimator. *Genetic epidemiology* 2016; **40**(4): 304-314; e-pub ahead of print 2016/04/12; doi 10.1002/gepi.21965.

9. Zhao Q, Wang J, Bowden J, Small D. Statistical inference in two-sample summary-data Mendelian randomization using robust adjusted profile score. *arXiv* 2018; **arXiv:1801.09652**: 1-59.

10. Hartwig FP, Davey Smith G, Bowden J. Robust inference in summary data Mendelian randomization via the zero modal pleiotropy assumption. *Int J Epidemiol* 2017; **46**(6): 1985-1998; e-pub ahead of print 2017/10/19; doi 10.1093/ije/dyx102.

11. Bowden J, Davey Smith G, Burgess S. Mendelian randomization with invalid instruments: effect estimation and bias detection through Egger regression. *Int J Epidemiol* 2015; **44**(2): 512-525; e-pub ahead of print 2015/06/08; doi 10.1093/ije/dyv080.

12. Yavorska OO, Burgess S. MendelianRandomization: an R package for performing Mendelian randomization analyses using summarized data. *Int J Epidemiol* 2017; e-pub ahead of print 2017/04/12; doi 10.1093/ije/dyx034.
